# RIPK3 promotes ASIC1a-mediated fibroblast-like synoviocyte migration and invasion via malate shuttle-driven mitochondrial respiration in rheumatoid arthritis

**DOI:** 10.7150/thno.113974

**Published:** 2025-08-11

**Authors:** Weirong Hu, Ke Wang, Yalu Dong, Yucai Xu, Jing Xing, Jianzhong Zhu, Jie Ding, Yingjie Zhao, Yayun Xu, Yuanzhi Cheng, Xiaoqing Peng, Renpeng Zhou, Wei Hu, Feihu Chen

**Affiliations:** 1 School of Pharmacy; Inflammation and Immune Mediated Diseases Laboratory of Anhui Province, Anhui Institute of Innovative Drugs, Anhui Medical University, Hefei 230032, China.; 2 Department of Clinical Pharmacology, the Second Affiliated Hospital of Anhui Medical University, Hefei, 230601, China.; 3 Shenzhen Institute of Translational Medicine, The First Affiliated Hospital of Shenzhen University, Shenzhen Second People's Hospital, China.; 4 Department of Orthopaedics and Rehabilitation, Yale University School of Medicine, New Haven, CT, USA.

**Keywords:** rheumatoid arthritis, fibroblast-like synoviocyte, ASIC1a, RIPK3, malate shuttle

## Abstract

**Objective:** Synovial fibroblast migration and invasion are critical contributors to the progression of rheumatoid arthritis (RA). Acidification of local joint tissue exacerbates RA progression, but the underlying mechanisms remain unclear. This study aimed to investigate the role of acid-sensitive ion channel ASIC1a and its mediator, the RIPK3-MDH1 axis, in regulating the migration and invasion of RA fibroblast-like synoviocytes (RA-FLSs).

**Methods:** The expression of ASIC1a, RIPK3, and MDH1 in synovial tissue from RA patients and arthritic mice was analyzed using immunofluorescence and Western blotting. RA-FLSs were stimulated with extracellular acidification (pH 6.8, mimicking local tissue conditions), and their migration and invasion were assessed via Transwell assays. The interaction between ASIC1a and RIPK3 was predicted using molecular docking and confirmed by co-immunoprecipitation (CO-IP). RIPK3^-/-^ mice were used to establish a collagen antibody-induced arthritis (CAIA) model. Pharmacological inhibitors of ASIC1a (PcTX1) and RIPK3 (GSK-872) were employed to evaluate their therapeutic effects on migration and invasion in vitro and arthritis progression in vivo using the collagen-induced arthritis (CIA) model. Bioinformatics analyses, along with glucose, ATP, NAD^+^ and NADH assays, and oxygen consumption rate (OCR) measurements, were conducted to investigate the regulation of mitochondrial respiration by the RIPK3-MDH1 axis.

**Results:** Extracellular acidification (pH 6.8) significantly enhanced the migration and invasion of RA-FLSs, effects that were abrogated by ASIC1a knockdown or pharmacological inhibition. ASIC1a activated RIPK3 through its kinase function, independent of its ion channel activity. RIPK3 activation promoted mitochondrial respiration and ATP production via MDH1-mediated malate shuttle activation. Furthermore, inhibition of the malate shuttle using Aminooxyacetic acid (Carboxymethoxylamine) hemihydrochloride (AOA) suppressed ASIC1a-mediated RA-FLSs migration and invasion. The RIPK3-MDH1 axis also maintained malate shuttle activity by enhancing glycolysis and glutamate metabolism through GLS1. Mechanistically, ASIC1a activated RIPK3, which in turn promoted MDH1-mediated malate shuttle activation, enhancing mitochondrial respiration and ATP synthesis, thereby driving RA-FLSs migration and invasion. In vivo, pharmacological inhibition of ASIC1a or RIPK3, as well as RIPK3 knockdown, significantly alleviated arthritis progression in CIA and CAIA mouse models.

**Conclusion:** The RIPK3-MDH1 malate shuttle drives RA-FLSs migration and invasion in RA. Activation of the ASIC1a-RIPK3-MDH1 axis enhances mitochondrial respiration and ATP synthesis in RA-FLSs, highlighting this pathway as a potential therapeutic target for RA.

## Introduction

Rheumatoid arthritis (RA) is a chronic autoimmune disease characterized by synovial proliferation, synovial inflammation, and pannus formation, which all contribute to cartilage destruction 1, 2. RA affects approximately 0.25%-1% of the global adult population 3. Advances in targeted therapies and early disease control have improved long-term prognosis, making early intervention and prevention of joint damage standard treatment strategies 4, 5. Fibroblast-like synoviocytes (FLSs) play a central role in joint destruction throughout both early and advanced RA stages 6, 7. These cells, located in the synovial membrane, maintain joint homeostasis under healthy conditions by producing extracellular matrix components and releasing matrix metalloproteinases (MMPs) 8, 9. However, in RA, FLSs become more invasive and adopt tumor-like traits, leading to articular cartilage damage 9. Thus, understanding the mechanisms of RA-FLSs migration and invasion is crucial for developing early treatment strategies and identifying potential therapeutic targets.

During the progression of RA, the joint microenvironment becomes acidified, primarily due to lactic acid accumulation from increased glycolysis in the synovium 10-12. Notably, studies have reported that the pH of synovial fluid in RA patients can drop significantly, with one study showing values as low as pH 6.0 and an average around 6.6, highlighting the extent of acidification 13-15. Moreover, synovial fluid pH has been shown to negatively correlate with RA disease activity, suggesting that lower pH levels are associated with greater disease severity 15. Our preliminary data indicate that an acidic medium (pH 6.0) promotes the migration and invasion of RA-FLSs. Mechanistically, extracellular acidification leads to intracellular Ca²⁺ elevation, which activates the Rac1 signaling pathway and upregulates the expression of matrix metalloproteinases MMP2 and MMP9 16. These changes facilitate the degradation of the extracellular matrix and enhance the invasive phenotype of RA-FLSs 17. A key mediator in this process could be acid-sensing ion channels (ASICs), which belong to the degenerin/epithelial sodium channel (DEG/ENaC) superfamily, are sensitive to extracellular protons, and comprise seven known isoforms: ASIC1a, ASIC1b, ASIC1b2, ASIC2a, ASIC2b, ASIC3, and ASIC4 17-19. Among these, ASIC1a is the most extensively studied, with well-characterized roles in the nervous system, bone tissue, and tumors, and its crystal structure has been resolved in chickens 19. ASIC1a is a proton-gated ion channel composed of over 500 amino acids, featuring a large extracellular domain, two transmembrane α-helices (TM1 and TM2), and intracellular N- and C-terminus 20. When the extracellular pH drops, hydrogen ions bind to the acid pocket of ASIC1a, triggering channel opening and allowing an inward flow of Na⁺ and Ca²⁺—a feature that distinguishes ASIC1a from other ASIC isoforms 20, 21. Functionally, ASIC1a contributes to acidosis-induced neuronal injury, pain signaling, and inflammatory responses. It is highly expressed in the central nervous system as well as in inflammatory conditions such as RA 19. Our previous studies have shown that ASIC1a is highly expressed in the synovium of rheumatoid arthritis (RA) patients and contributes to inflammation, prompting us to investigate its role in regulating the migration and invasion of RA-FLSs 22. We further found that ASIC1a mediates the migration and invasion of RA-FLSs under acidic conditions (pH 6.0), potentially through Ca²⁺ influx and subsequent activation of the Rac1 signaling pathway 16. Similar acid-driven roles have been observed in glioma cells, where ASIC1a facilitates migration and invasion in response to weak acid-induced changes 23. Taken together, these findings highlight ASIC1a as a critical mediator in RA pathogenesis, particularly within the acidic joint microenvironment. However, further studies are needed to fully elucidate its mechanisms of action in RA.

Metabolism encompasses the biochemical reactions that convert a variety of nutrients into small molecule metabolites within cell biology 24. These transformations produce energy, redox equivalents, and macromolecules such as proteins and lipids, which are essential for cell survival and function 25. A number of studies have shown that the metabolism of RA-FLSs is significantly altered and causes unavoidable damage to the joints. Metabolic pathways such as glycolysis, amino acid metabolism, and oxidative phosphorylation (OXPHOS) are significantly altered in RA-FLSs compared to osteoarthritis fibroblast-like synoviocytes (OA-FLSs) 12, 26. Alterations in the metabolic preferences of RA-FLSs significantly impact their migratory and invasive capabilities 27. Importantly, the acidified extracellular microenvironment serves as a key driver of metabolic reprogramming. Research has shown that extracellular acidification enhances fatty acid oxidation and mitochondrial respiration in tumor cells, thereby supporting cell survival 28. Notably, recent findings indicate that ASIC1a also regulates mitochondrial respiration and ATP production as a metabolic regulator 29, 30. However, it remains unclear whether ASIC1a mediates metabolic reprogramming in the extracellular acidified microenvironment to regulate RA-FLS migration and invasion.

In this study, we explored the role of ASIC1a in extracellular acidification-mediated migration and invasion of RA-FLSs by pharmacologically blocking or genetically disrupting ASIC1a and further investigated its function as a metabolic regulator in acidification-induced metabolic alterations using shRNA interference and metabolomic profiling. The results demonstrated that extracellular acidification enhances RA-FLSs migration and invasion by altering their metabolic profile, while ASIC1a suppression inhibited these effects in vitro and attenuated RA progression.

## Materials and Methods

### Human samples

RA and normal synovium samples were obtained from patients at the First Affiliated Hospital of Anhui Medical University. RA patients underwent knee replacement surgery and were diagnosed according to the standards set by the American College of Rheumatology. Normal synovium samples were donated by patients who had undergone traffic accidents and had no history of RA. These trauma patients also had no history of osteoarthritis (OA), other inflammatory joint diseases, or significant knee joint injuries, as confirmed by clinical records and histological assessment. These procedures were approved by the Clinical Research Ethics Committees of Anhui Medical University (approval number: YX2022064). Detailed information about the donors is listed in Table S1.

### Cell culture and treated

Under sterile conditions, we quickly washed the synovial tissue once with 75% alcohol and subsequently performed PBS washes twice. The synovial tissue was minced in PBS to roughly 1 mm^3^ in size. Then, the tissue was evenly spread on the wall of cell culture flasks using a Pasteur pipette and the cell flasks were supplemented with DMEM/ high-glucose medium (Vivacell, China) containing 20% FBS (Biochannel, China) and 1% penicillin/streptomycin (Beyotime, China). The culture flasks were placed upright in a 37 ℃, 5% CO_2_ and 95% humidity incubator for 6 hours and then gently flattened. Passaging was performed after the formation of synovial fibroblast colonies. Only RA-FLSs at passages 4, 5, 6, or 7 were used across all experiments to ensure consistency in cell behavior and phenotype 31, 32.

HEK-293T cells were cultured in DMEM/high-glucose medium supplemented with 10% FBS, 1% penicillin/streptomycin, and 25 mM HEPES to maintain pH stability under acidic conditions. These cells were selected due to their rapid proliferation and high tolerance to extracellular acidification, which allowed us to obtain sufficient cytoplasmic protein and perform subcellular localization analysis under pH 6.8 conditions.

Acidified medium was prepared by adding 1N HCL to DMEM/high-glucose Medium with HEPES (bioship, China) containing 20% FBS and 1% penicillin/streptomycin. The day before treating the cells, the medium was tested using a pH meter (shlei-ci, PHB-4), and the acidified medium, which had a stable pH 6.8 ± 0.05, was subsequently placed in an incubator for 24 h to induce buffering. The pH value stabilized at 6.8 ± 0.05 using a pH meter before using the acidified medium on the next day. Fresh acidified medium was changed every 24 h while treating the cells, and as a control the non-acidified group was also changed with fresh medium at the same time. Specific information on the inhibitors and drugs used for cells-treatment was listed in Table S2.

### Transwell assay

The effect of extracellular acidification on the migratory and invasive ability of RA-FLSs was detected by Transwell. Briefly, we treated RA-FLSs in six-well plates, including treatment with acidified medium and various inhibitors such as PcTX1 and GSK872. After treating RA-FLSs with acidified medium with or without the addition of inhibitors, we digested and resuspended the cells. The cell suspension was subsequently diluted to 2.0×10⁵ cells per milliliter using medium containing 20% FBS. A cell suspension (200 µL) was seeded in the upper chamber of the Transwell insert (24-well insert; 8 µm pore size; Corning, USA). Then, 600 µL of medium containing 20% FBS was added to the lower chamber. After 8 hours in the incubator, the cells had attached to the polycarbonate membrane. The medium in the upper chamber was changed to serum-free acidified medium (with or without inhibitors), and the medium in the lower chamber was changed to acidified medium containing 20% FBS (with or without inhibitors) to act as a chemoattractant. After 36 h in the incubator, we gently removed the non-migrating cells from the top of the membrane with a PBS-soaked cotton swab. The upper chamber was immersed in methanol to fix the migrating cells for 20 min, followed by staining with 0.1% crystal violet for 20 min. Excess crystal violet was washed away with PBS. Three random images of the upper chamber were taken under a 100x microscope, and the average number of migrating cells was recorded.

The invasion test was performed in the same way as described above, using an upper chamber coated with 60 µL of Matrigel solution (0.5 µg/µL, Corning, USA) on the membrane of the upper chamber.

### Western blot

For extraction of total protein, cells were lysed on ice by adding 200 µL of RIPA buffer (Beyotime, China) containing 1% protease inhibitor cocktail (HY-K0010, MCE) and phosphatase inhibitor cocktail (HY-K0021, MCE) per well in a six-well plate. Cell lysates were collected and centrifuged at 12,000 rpm for 15 minutes at 4 ℃. The extraction of cellular cytoplasmic proteins was performed using a cytoplasmic protein extraction kit (Beyotime, China). Protein concentrations were quantified by BCA protein assay (Beyotime, China) and subsequently diluted using SDS-PAGE sample loading buffer.

Protein samples obtained using the method described above were electrophoresed in 8-15% SDS-PAGE gels and subsequently transferred to polyvinylidene fluoride membranes. Membranes were blocked with 5% non-fat milk and then incubated with primary antibodies (Table S3) at 4 ℃ overnight. The TBST-rinsed membrane was then incubated with the HRP-conjugated secondary antibody for 2 h at room temperature. The enhanced chemiluminescence solution (Affinity, USA) was uniformly applied to the membrane and detected using an automated chemiluminescence imaging analysis system (Tanon, China). The IOD values of the protein bands were quantified using ImageJ software.

### Quantitative real-time PCR

Total RNA from RA-FLSs was extracted using TRIzol (Accurate Biology, China) and reverse transcribed into cDNA using 5×Evo M-MLV RT Master Mix (Accurate Biology, China). Samples were analyzed by qRT-PCR using 2×SYBR Green Pro Taq HS Premix (Accurate Biology, China). Data were normalized to GAPDH as an endogenous control and transcript levels were analyzed by the 2^-△△Ct^ method. The primer sequences are listed in Table S4.

### Immunofluorescence staining

Tissue sections were deparaffinized with xylene and soaked in graded ethanol. Subsequently, the sections were permeabilized using 0.3% Triton X-100 (Solarbio, China). After blocking with 1% BSA, the tissue sections were incubated with the primary antibody at 4 ℃. The following day, the sections were stained with fluorescent secondary antibodies (ZSGB-Bio, China). Cell nuclei were counterstained with DAPI (Beyotime, China). The samples were then imaged using a fluorescence microscope. A catalog of immunofluorescent antibodies is attached in Table S3.

### Bioinformatic data mining

Differential gene expressions between RA synovium and healthy synovium were analyzed using the GSE77298, GSE48780 and GSE1919 datasets from the Gene Expression Omnibus (GEO) database. Differentially expressed genes (DEGs) were presented as heatmaps and GSEA enrichment analysis (GSEA v4.4.4).

### Calcium imaging

Wash acidified medium-treated RA-FLSs (either adhered to six-well plates or digested into a cell suspension) with D-Hanks' solution. Next, incubate the cells at 37 ℃ for 30 min with Fluo-4 AM (5 µM; Beyotime, China). The cells were then imaged using a fluorescence microscope, or the fluorescence intensity was measured using flow cytometry (excitation at 488 nm).

### ASIC1a gene knockdown

RA-FLSs were infected with ASIC1a shRNA lentiviral vector (Table S5) for 48 h. Cells were screened for 72 h post-infection using 4 µg/ml puromycin. Subsequently, the cells were continuously cultured using 300 ng/ml puromycin. The knockdown efficiency of ASIC1a was characterized by Western blot and qPCR.

### MTT assay

The cells were diluted to 5000 cells/100 µL. 100 µL of cell suspension was seeded into 96-well plates. The MTT solution (Beyotime, China) was added after treatment of cells with acidified medium or drugs, followed by incubation at 37 ℃ for 4 h. Subsequently, the medium was aspirated, and 100 µL of DMSO solution (Solarbio, China) was added and mixed for 10 min on a horizontal shaker protected from light. Finally, absorbance values were measured at 490 nm using a multifunctional microplate reader.

### CO-immunoprecipitation (CO-IP)

Cells were lysed using a weak lysis buffer. Ten percent of the cell lysate was retained as input. The remaining lysate was incubated with anti-ASIC1a antibody and IgG, respectively, for one hour on a vertically rotating shaker. Subsequently, protein A/G was added, and the mixture was rotated at 4 ℃ overnight. After washing the immune complexes six times with lysis buffer, they were eluted using SDS-PAGE loading buffer and heated at 100 ℃ for 10 minutes. Protein samples were then analyzed by Western blot.

### Glucose, ATP, NAD^+^, NADH and BCA protein assay

Cells were seeded in six-well plates (5×10⁴ cells/well) and washed twice with PBS after treatment with acidified medium and inhibitors. Next, cells were collected using the Glucose, ATP, NAD^+^, NADH kit's specified lysates. The intracellular Glucose, ATP, NAD^+^, NADH assay was performed according to the manufacturer's instructions. Finally, the obtained intracellular Glucose, ATP, NAD^+^, and NADH were standardized and quantified in terms of protein concentration for each group. Specific information on the kits is listed in Table S6.

### Oxygen consumption measurements

After treating RA-FLSs in six-well plates, cells were diluted using plating medium and seeded onto poly-D-lysine-coated 24-well plates (Agilent, USA) at 2×10⁴ cells/well. The cultures were incubated in an incubator for 24 h. 24 h before the assay, the probe plate was immersed in the XF calibration solution (Agilent, USA) and placed in an incubator with CO₂. On the day of the experiment, the medium in the cultures was replaced with an assay solution containing glucose, glutamine, and pyruvate, and placed in an incubator without CO₂ at 37 ℃ for 1 h. At the same time, reagents (Oligomycin 1.5 µM, FCCP 2 µM, Rot/AA 0.5 µM) were added to the port of the probe plate, and the processed probe plate was placed into the hippocampal respirometer for calibration. The assay instrument used is the Agilent Seahorse XFe24, and the final data were exported by Wave (Agilent, USA).

### Inducing and treating the collagen induced arthritis (CIA) model

Male DBA 1/J mice were obtained from Charles River Laboratories. Mice were injected with 100 µL of 0.25 mg of complete Freund's adjuvant (Sigma) emulsified with chicken type II collagen at the base of the tail on days 1 and 21, respectively. Clinical scores for each paw were assessed every three days by the same independent blind observers, with a score of 0-4 recorded for each paw. On day 30 of the CIA model, the success rate of the model was assessed, and the failed models' mice were excluded. Mice were randomly divided into 6 groups: ctrl, CIA model, CIA+PcTX1 (intraarticularly administered, 3 µg/kg/3 days), CIA+GSK872 (i.p. 1 mg/kg/3 days), CIA+Amiloride (i.g. 2 mg/kg/3 days), and CIA+MTX (i.g. 1 mg/kg/3 days). On day 52, mice were euthanized, and blood and ankles were collected for subsequent studies. All animal experiments were conducted in accordance with institutional guidelines and approved by the Animal Ethics Committee of Anhui Medical University (approval number: LLSC20220383).

### CAIA

On day 0, 5 mg of type II collagen antibody was injected intraperitoneally into both RIPK3 conventional knockout mice and wild-type C57BL/6NCya mice. On day 3, LPS was administered intraperitoneally to enhance the immunization response. Arthritis scores were observed and recorded daily until day 15, when the mice were euthanized.

Arthritis scores were assessed on both hind paws by the same independent blind observers. The scoring system included four grades: 0, no localized redness or swelling; 1, swelling in the knuckle joints; 2, mild swelling in the ankle joints; 3, severe swelling in the entire paw; and 4, stiffness or deformation of the paw. All animal experiments were conducted in accordance with institutional guidelines and approved by the Animal Ethics Committee of Anhui Medical University (approval number: LLSC20220383).

### Micro-CT

Bone mass of mouse ankle joints was analyzed using a Skyscan 1772 Micro-21 CT system. Data obtained from the CT system were reconstructed using NRecon, and samples were drawn by CTvox4 software (Skyscan Bruker, Tuckson, USA). Bone morphology parameters analyzed included bone density (BMD), bone volume percentage (BV/TV), bone surface to tissue volume (BS/TV), trabecular thickness (Tb. Th), trabecular number (Tb.N), and trabecular separation (Tb.Sp), and others.

### Histological analysis of arthritic joint

The entire hind paws of the mice were fixed in 10% formalin for 48 hours, followed by decalcification in 10% EDTA for 4 weeks. After trimming, the tissues were embedded and sectioned. The tissue sections were then stained with H&E and Safranin O and assessed for histopathology.

Joint inflammation was scored based on H&E staining as follows: 0 = normal; 1 = few inflammatory cells around the joints; 2 = mild infiltration; 3 = moderate infiltration. Bone resorption was scored based on H&E staining as: 0 = normal; 1 = mild (small, inconspicuous resorption areas under low magnification); 2 = moderate (more resorbed areas in the bone trabeculae or cortical bone, with clear lesions under low magnification); 3=moderate (obvious resorption of trabecular and cortical bone, without full thickness defects in the cortex; loss of some trabeculae; lesions apparent on low magnification); and 4 = significant (full-thickness cortical bone defects with prominent changes in the trabeculae).

Cartilage depletion was scored according to Safranin O staining as: 0 = no cartilage damage (complete Safranin O staining); 1 = localized erosion; 2 = extensive erosion; 3 = severe erosion; and 4 = total depletion. Histological scoring was performed in a blinded manner, following the methodology outlined in Doody's article 33.

### ELISA

Serum levels of IL-1β, IL-6, TNF-α, and malic acid in mice were measured according to the manufacturer's instructions for the IL-1β, IL-6, TNF-α, and malic acid precoated ELISA kits. ELISA kit information is attached in Table S6.

### Statistical analysis

Statistical analyses were conducted using GraphPad Prism 8.0.0. Each data set comprised a minimum of three independent sample replicates, and results are expressed as mean ± standard error of the mean (SEM). Differences between two groups were assessed using Student's t-test, while comparisons among multiple groups were conducted using one-way or two-way ANOVA. A *P*-value of less than 0.05 was considered statistically significant.

## Results

### ASIC1a mediates acidification-induced migration and invasion of RA-FLSs

**Figure 1:**
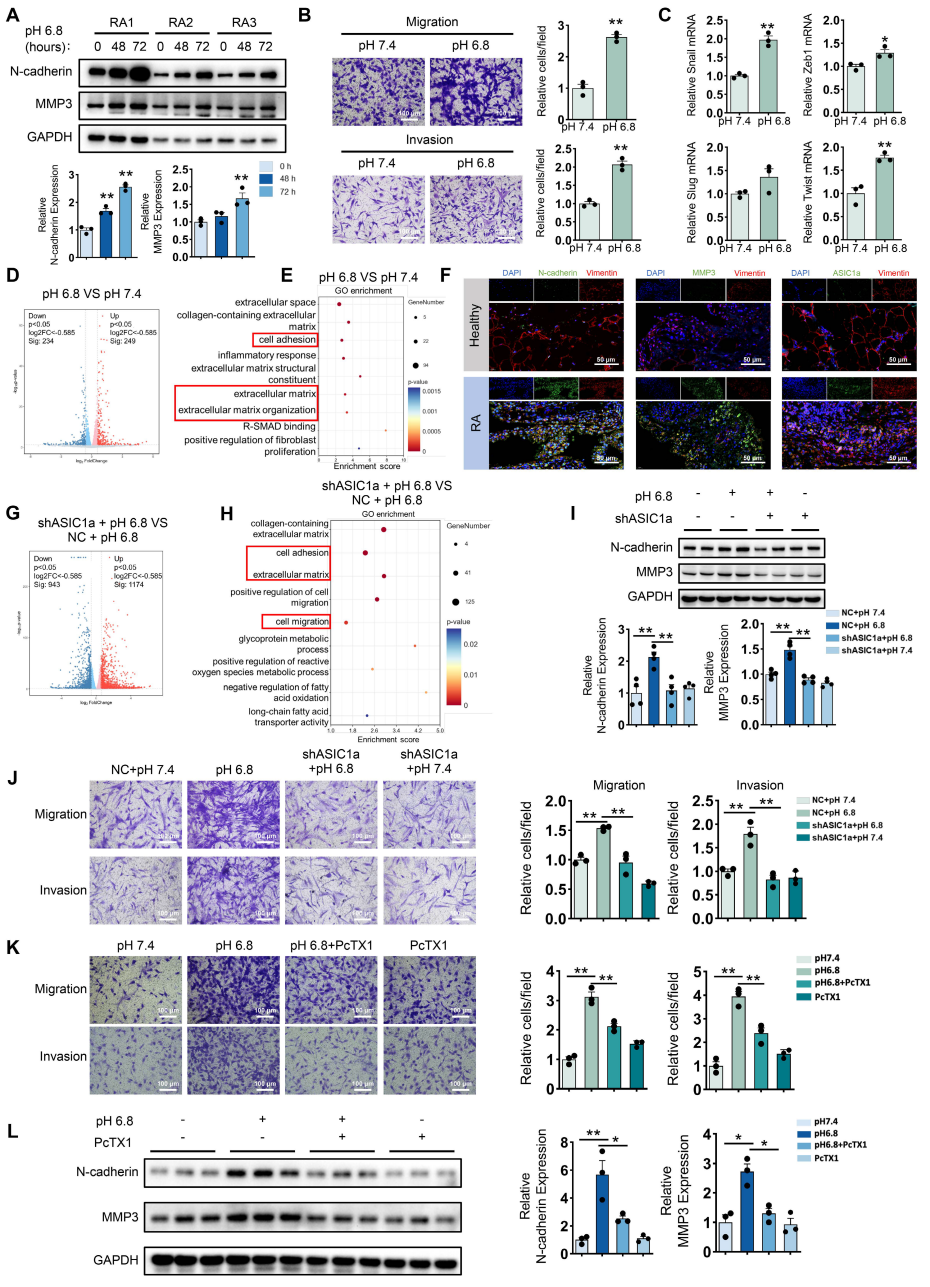
** ASIC1a is involved in extracellular acidification-induced migration and invasion of RA-FLSs.** (A) Western blot analysis of N-cadherin and MMP3 in pH 6.8 medium-treated RA-FLSs (n = 3). (B) Transwell assay measuring the migration and invasion ability of RA-FLSs (n = 3). (C) The relative mRNA expression levels of Snail, Zeb1, Slug and Twist in RA-FLSs (n = 3). (D, E) Volcano plots show the DEGs of RA-FLSs treated with pH 6.8 medium, and GO enrichment analysis was performed to analyze the biological processes associated with these DEGs. (F) Immunofluorescence analysis of N-cadherin, MMP3 and ASIC1a expression in the synovium of RA patients. (G, H) Volcano plot showing DEGs in RA-FLSs after ASIC1a knockdown, and GO enrichment analyses showing biological processes associated with these DEGs. (I) Western blot analysis of N-cadherin and MMP3 protein expression in RA-FLSs (n = 4). (J, K) Transwell assayed the effect of the migratory and invasive capacities of RA-FLSs (n = 3). (L) Western blot analysis of N-cadherin and MMP3 in RA-FLSs (n = 3). The data are presented as the means ± SEM, with the data being analyzed by unpaired two-tailed t-test (two groups) or one-way ANOVA (multiple groups). (*p < 0.05, **p < 0.01).

To investigate the effect of extracellular acidification on the migration and invasion of RA-FLSs, we first employed a pH 6.8 medium to mimic the acidified intra-articular microenvironment in the treatment of primary FLSs isolated from the synovial tissue of RA patients. Western blot analysis revealed that the expression levels of N-cadherin and MMP3 increased with prolonged exposure to the acidified medium (Figure 1A). Next, the Transwell assay demonstrated that treatment with pH 6.8 medium for 72 h significantly enhanced the migration and invasion capabilities of RA-FLSs (Figure 1B). Notably, combined with MTT assay results, we found that pH 6.8 medium promoted migration and invasion of RA-FLSs without affecting their proliferative ability (Figure S1A). In addition, Western blot analysis of Bax and Caspase-3 expression, along with Annexin V-FITC/PI staining, showed that pH 6.8 did not induce apoptosis in RA-FLSs (Figure S1B-C). Furthermore, treatment with pH 6.8 increased the expression of epithelial-mesenchymal transition transcription factors (EMT-TFs), such as Snail, Zeb1, and Twist, in RA-FLSs (Figure 1C). These findings were consistent with the synovial characteristics observed in RA patients. In support of this, Gene Set Enrichment Analysis (GSEA) of the GEO dataset (GSE77298) revealed significant enrichment of the fibroblast migration pathway in RA synovium (Figure S2A). Additionally, both Western blot and immunofluorescence staining confirmed that N-cadherin and MMP3 levels were elevated in RA synovium and RA-FLSs compared to healthy controls (Figure 1F and Figure S2B). Transcriptomic analysis showed that differentially expressed genes between pH 7.4- and pH 6.8-treated RA-FLSs were significantly enriched in pathways associated with migration and invasion (Figure 1D-E). Collectively, these data suggest that extracellular acidification (pH 6.8 medium) effectively mimics the joint microenvironment in vitro and significantly enhances the migration and invasion of RA-FLSs.

To explore whether ASIC1a is involved in the migration and invasion of RA-FLSs promoted by extracellular acidification environment (pH 6.8), we first examined the expression of ASIC1a in synovium. Western blot analysis showed that ASIC1a expression was significantly upregulated in synovial fibroblasts isolated from RA patients compared to those from healthy individuals (Figure S2C). Consistently, analysis of the GEO database and immunofluorescence staining further confirmed that ASIC1a expression was markedly elevated in RA synovial tissues relative to healthy controls (Figure S2D and Figure 1F). Furthermore, ASIC1a expression showed a strong positive correlation with the expression of MMP family members and EMT-related genes (Figure S2E). To investigate the role of ASIC1a in pH 6.8-induced migration and invasion of RA-FLSs, we knocked down its expression using shASIC1a (Figure S2F-G). Transcriptional analysis indicated that ASIC1a was involved in the migration and invasion of RA-FLSs induced by pH 6.8 medium (Figure 1G-H). Moreover, Western blot analysis showed that the enhancement of N-cadherin and MMP3 by pH 6.8 medium was reversed following ASIC1a knockdown (Figure 1I). Additionally, ASIC1a knockdown dramatically reduced the migration and invasion capacity of RA-FLSs treated with pH 6.8 medium (Figure 1J). Similarly, blocking ASIC1a with PcTX1, a specific inhibitor, also reduced pH 6.8 medium-induced migration and invasion of RA-FLSs, as well as the expression levels of N-cadherin and MMP3 (Figure 1K-L). Taken together, these results demonstrate that ASIC1a mediates pH 6.8 medium-induced migration and invasion, thus contributing to the progression of RA.

### ASIC1a enhances migration and invasion of RA-FLSs by promoting mitochondrial respiration in a weak extracellular acidification

**Figure 2:**
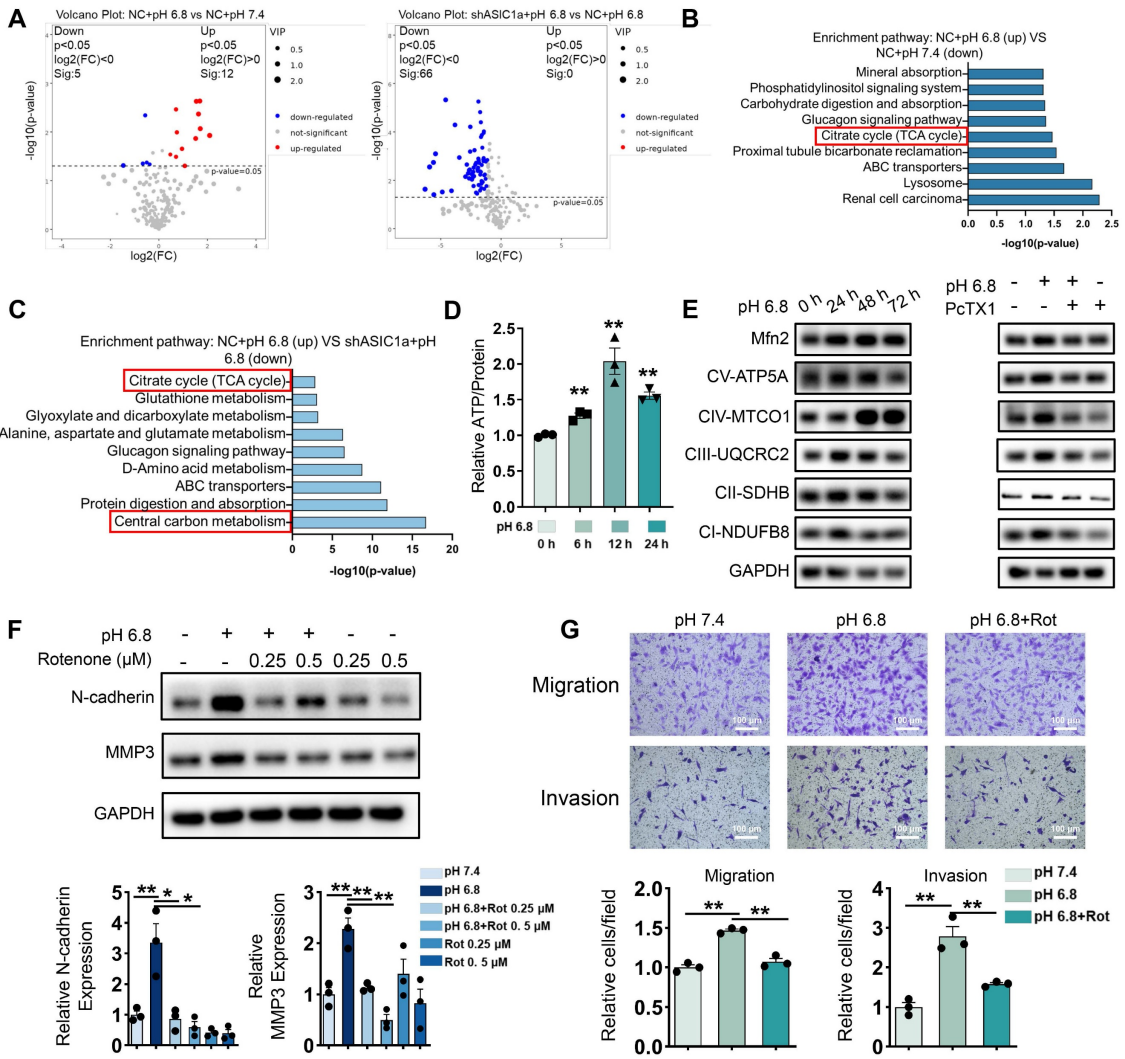
** ASIC1a promotes migration and invasion of RA-FLSs by enhancing mitochondrial respiration.** (A) A volcano plot showing the differential metabolites identified through GC-MS/MS analysis. (B, C) GO enrichment analysis of the biological processes of metabolites differing between pH 6.8 vs pH 7.4, pH 6.8 vs shASIC1a + pH 6.8. (D) Detection of ATP levels in RA-FLSs (n = 3). (E) Western blot to detect the effects of pH 6.8 medium or ASIC1a inhibition on the expression of Mfn2 and mitochondrial respiration biomarkers, respectively (n = 3). (F) Effect of Rotenone, an inhibitor of mitochondrial respiration, on the expression of N-cadherin and MMP3 as detected by Western blot (n = 3). (G) Transwell assay the effect of the migratory and invasive capacities of RA-FLSs in pH 6.8 medium treated with or without Rotenone (n = 3). The data are presented as the means ± SEM, with the data being analyzed by unpaired two-tailed t-test (two groups) or one-way ANOVA (multiple groups). (*p < 0.05, **p < 0.01).

As shown in Figure 1H, ASIC1a is involved in multiple metabolic processes. To further investigate the metabolic alterations in pH 6.8-treated RA-FLSs regulated by ASIC1a, we conducted an untargeted metabolomics study using both GC-MS/MS and LC-MS/MS. Metabolomics analysis by GC-MS/MS revealed that ASIC1a regulates key energy metabolic pathways, including central carbon metabolism and TCA cycle (Figure 2A-C). Central carbon metabolism is a crucial pathway for ATP production, which plays a vital role in sustaining tumor cell migration and invasion 34. Transcriptomic analysis further supported these findings. We identified genes with an absolute log ratio of (shASIC1a + pH 6.8 vs NC + pH 6.8) / (NC + pH 6.8 vs pH 7.4) ≤ 2 that were significantly regulated by ASIC1a and analyzed their transcriptional profiles (Figure S3A). KEGG enrichment analysis showed that the genes regulated by ASIC1a were significantly enriched in the central carbon metabolism pathway (Figure S3B). Moreover, GSEA analysis of the GSE77298 and GSE1919 datasets revealed that synovial membranes from RA patients were significantly enriched in catabolic and biosynthetic processes, both of which are closely associated with ATP acquisition (Figure S3C).

The results obtained using GC-MS/MS, both individually and in combination with LC-MS/MS, were consistent with these findings (Figure S4A-E). Similarly, GEO data analysis revealed upregulation of genes related to glycolysis, fatty acid oxidation, and amino acid metabolism in the synovium of RA patients (Figure S4F-G). These metabolic pathways are interconnected with central carbon metabolism, further suggesting that our in vitro extracellular acidification model effectively replicates the altered energetic profile of RA-FLSs in the joint. Furthermore, pH 6.8 treatment enhanced intracellular ATP levels, which were inhibited by the ASIC1a-specific inhibitor PcTX1 (Figure 2D and Figure S5A). These results suggest that ASIC1a facilitates ATP acquisition in RA-FLSs under extracellular acidification conditions, thereby supporting their migration and invasion.

Moreover, these findings imply that RA-FLSs require increased ATP production during RA progression. Since oxidative phosphorylation (OXPHOS) is the most efficient ATP production pathway, we assessed mitochondrial respiration biomarkers in pH 6.8-treated RA-FLSs via Western blot analysis. Both OXPHOS and the mitochondrial fusion protein Mfn2 were significantly upregulated in pH 6.8-treated RA-FLSs, which is consistent with the idea that enhanced mitochondrial fusion promotes efficient OXPHOS, and this effect was reversed by PcTX1 (Figure 2E, and Figure S5B-C). Additionally, GEO data further demonstrated elevated mitochondrial respiratory gene expression in the synovium of RA patients compared to healthy donors (Figure S5D-E). Importantly, the increased expression of N-cadherin and MMP3, as well as the enhanced migratory and invasive abilities of RA-FLSs induced by pH 6.8, were reversed by the mitochondrial respiration inhibitor rotenone (Figure 2F-G). These results confirm that ASIC1a promotes RA-FLSs migration and invasion by mediating metabolic reorganization in the extracellularly acidified microenvironment, likely through enhanced mitochondrial respiration and ATP production.

### RIPK3 involved in ASIC1a-driven mitochondrial respiration in RA-FLSs to promote migration and invasion

**Figure 3:**
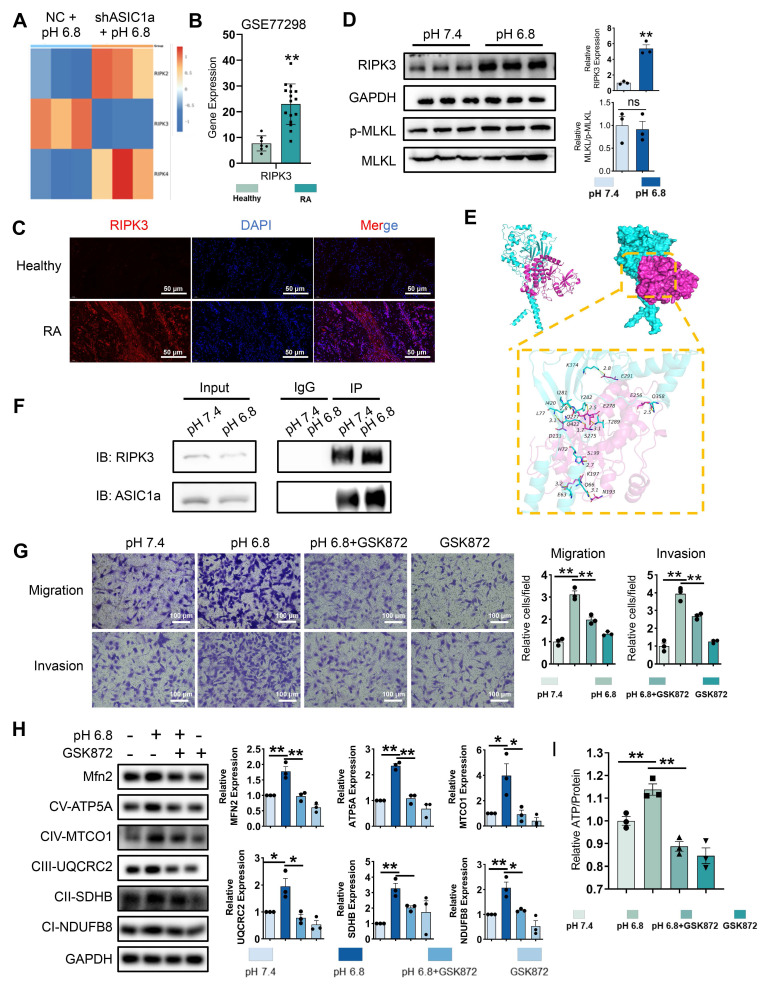
**RIPK3 is involved in ASIC1a-mediated enhancement of mitochondrial respiration, migration and invasion in RA-FLSs.** (A) Heatmap showing the effect of ASIC1a knockdown on the expression of the RIPK family. (B) Expression characterization of RIPK3 in GSE77298. (C) Immunofluorescence analysis of RIPK3 expression in the synovium of RA patients. (D)Western blot analysis of N-cadherin, MMP3, MLKL and p-MLKL in RA-FLSs (n = 3). (E) 3D binding structure of ASIC1a and RIPK3 determined via molecular modeling and docking studies. (F) The interaction between ASIC1a and RIPK3 in RA-FLSs was analyzed by co-immunoprecipitation. (G) Transwell assay the effect of the migratory and invasive capacities of RA-FLSs treated with or without GSK872 (n = 3). (H) Western blot to detect the expression of Mfn2 and mitochondrial respiration biomarkers treated with or without GSK872 (n = 3). (I) Detection of ATP levels in RA-FLSs (n = 3). The data are presented as the means ± SEM, with the data being analyzed by unpaired two-tailed t-test (two groups) or one-way ANOVA (multiple groups). (*p < 0.05, **p < 0.01).

The role of ASIC1a-mediated Ca²⁺ influx in the pathogenesis of various diseases has been extensively studied 19. We examined Ca²⁺ levels in RA-FLSs treated with pH 6.8 medium. However, fluorescence and flow cytometry showed no significant increase in Ca²⁺ levels in pH 6.8-treated RA-FLSs compared to controls (Figure S6A-B), suggesting that the role of ASIC1a functioning as an ion channel does not play a dominant part in the context of a pH 6.8 environment. Beyond mediating calcium influx, ASIC1a also activates the RIPK family, including RIPK1 and RIPK3, to promote necroptosis and drive RA progression. Notably, the RIPK family is also implicated in cell migration 35. To investigate whether the RIPK family contributes to ASIC1a-mediated migration and invasion of RA-FLSs, we first analyzed the expression of RIPK family members in the transcriptome. ASIC1a knockdown resulted in decreased RIPK3 expression (Figure 3A). Similarly, GSE77298 dataset analysis revealed increased RIPK3 expression in RA synovium (Figure 3B). Western blot analysis revealed that the protein level of RIPK3 was significantly elevated in primary synovial fibroblasts derived from RA patients compared to those from healthy donors (Figure S2C). This was further supported by immunofluorescence staining, which showed higher RIPK3 expression in RA synovial tissue relative to healthy controls (Figure 3C). Moreover, Western blot analysis demonstrated that RIPK3 levels were also increased in RA-FLSs following treatment with pH 6.8 medium (Figure 3D). However, the expression levels of MLKL and p-MLKL remained unchanged under acidic conditions (Figure 3D), suggesting that RIPK3 upregulation induced by pH 6.8 occurs independently of necroptosis.

To investigate the interaction between ASIC1a and RIPK3, we performed molecular docking to identify potential binding sites between the two proteins (Figure 3E). Co-IP further confirmed enhanced binding of ASIC1a to RIPK3 following pH 6.8 treatment (Figure 3F). Additionally, PcTX1 inhibited the pH 6.8-induced expression and phosphorylation of RIPK3, indicating that ASIC1a regulates both the expression and phosphorylation of RIPK3 (Figure S6C). Given the potential role of RIPK3 in migration and invasion, we used GSK872, a specific RIPK3 inhibitor, to suppress RIPK3 expression and assess its effect on pH 6.8-induced RA-FLSs migration and invasion. As expected, GSK872 effectively inhibited RA-FLSs migration and invasion (Figure 3G). Furthermore, Western blot analysis showed that GSK872 not only suppressed acidification-induced RIPK3 and p-RIPK3 expression but also reversed N-cadherin and MMP3 expression (Figure S6D). GSK872 also inhibited mitochondrial respiration markers and Mfn2 (Figure 3H) and reversed the ATP increase induced by pH 6.8 (Figure 3I). These results suggest that ASIC1a, through its interaction with RIPK3, enhances mitochondrial respiration in RA-FLSs under weak extracellular acidification, thereby promoting migration and invasion.

**Figure 4:**
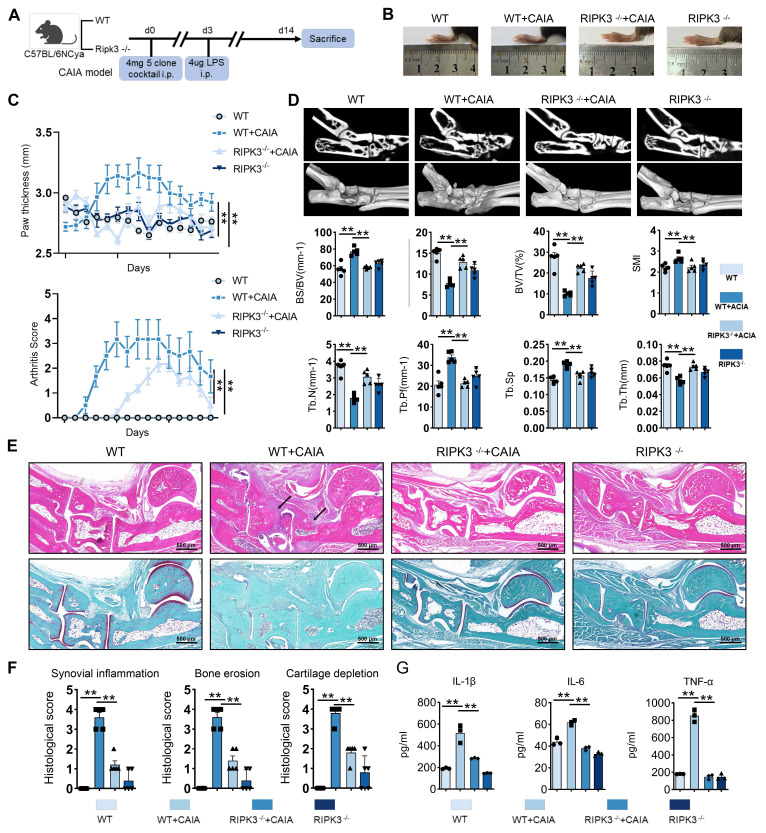
**Knockout of RIPK3 attenuates the progression of RA in the CAIA model.** (A) RIPK3 knockout mice were used to construct the CAIA models (n = 6). (B, C,) Compared with the CAIA model using the same wild-type littermate, the CAIA model of RIPK3 knockout mice had lower paw thickness and arthritis index (n = 6). (D) Micro-CT analysis for the ankles of mice in the indicated groups and quantitative analysis (n = 5). (E) H&E staining shows that RIPK3 knockout significantly inhibits the overall pathogenic destruction and synovial proliferation, migration and invasion in the joint of CIA mice (black arrows) and Safranin O staining demonstrate that RIPK3 knockout significantly represses destruction of bone and cartilage in the joint of CIA mice. (F) Histological analysis of synovial inflammation scored, Bone erosion score, and cartilage depletion (n = 5). (G) RIPK3 knockout alleviates CAIA-induced systemic inflammation. The data are presented as the means ± SEM, with the data being analyzed by one-way ANOVA or two-way ANOVA. (*p < 0.05, **p < 0.01).

RIPK3 knockout mice were used to establish the CAIA model to investigate RIPK3's role in RA pathogenesis (Figure 4A). Compared to wild-type (WT) CAIA mice, joint swelling in the hind limbs was significantly reduced in RIPK3^-/-^ CAIA mice (Figure 4B-C). The arthritis score in RIPK3^-/-^ mice was notably lower than in wild-type CAIA mice (Figure 4C). Micro-CT analysis revealed bone erosion and rough surfaces in wild-type CAIA mice, while RIPK3 knockout mitigated bone destruction (Figure 4D). H&E, Safranin O, and TRAP staining further showed that RIPK3^-/-^ alleviated synovial proliferation, invasion, and cartilage destruction (Figure 4E-F, and Figure S7A). Additionally, ELISA results demonstrated reduced inflammation in RIPK3^-/-^ CAIA mice (Figure 4G). These findings indicate that RIPK3 is involved in RA-FLSs migration and invasion induced by the extracellular acidified microenvironment in vitro, and in vivo experiments confirmed its role in RA progression in animal models.

### RIPK3/ASIC1a enhanced malate shuttle-mediated mitochondrial respiration, migration, and invasion in RA-FLSs

**Figure 5:**
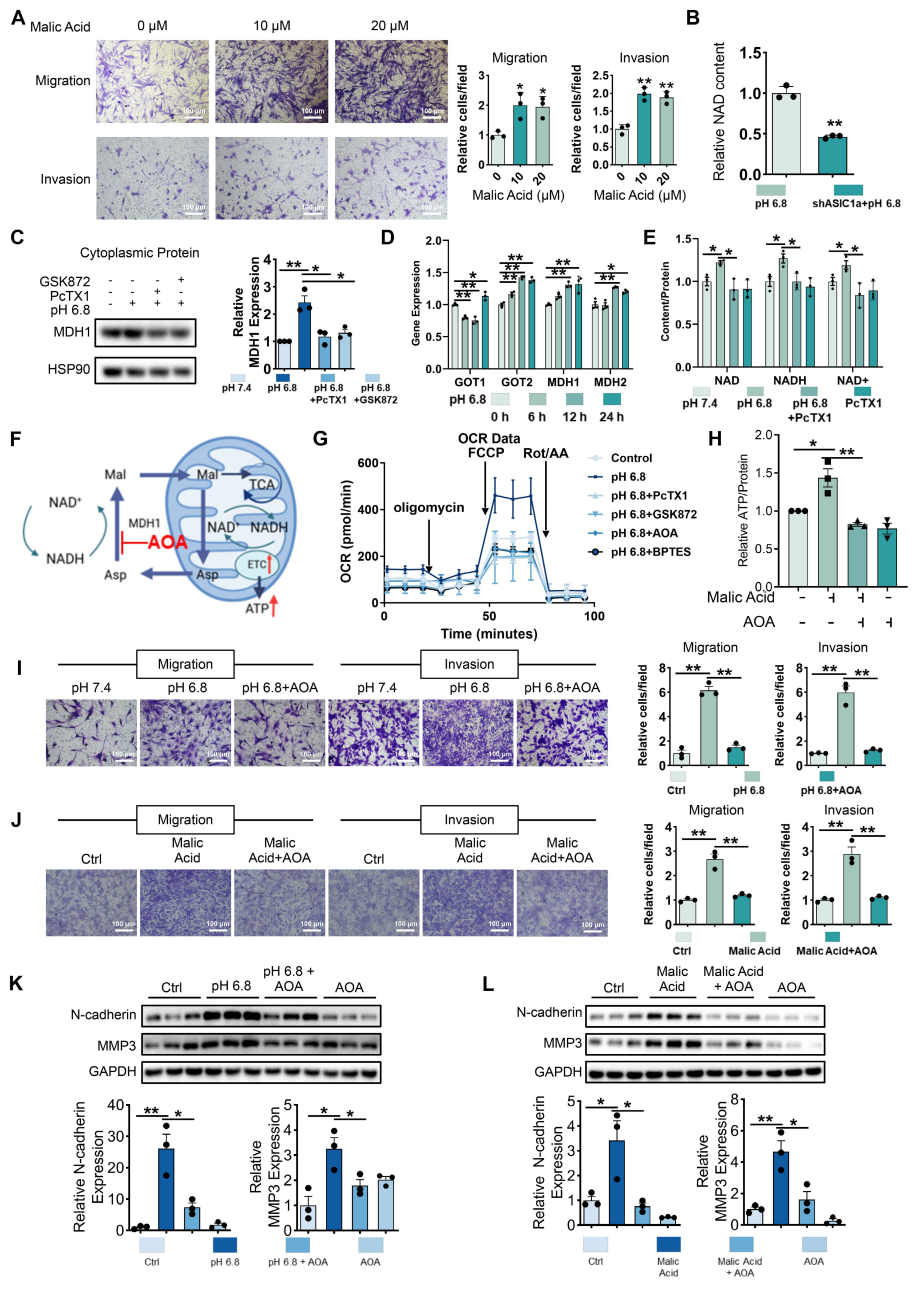
** Malate shuttles promote mitochondrial respiration, migration and invasion of RA-FLSs.** (A) Exogenous malic acid promotes migration and invasion of RA-FLSs (n = 3). (B) Metabolomics showed that ASIC1a knockdown inhibited NAD levels in RA-FLSs. (C) Western blot detected MDH1 in cytoplasmic proteins of HEK-293T cells (n = 3). (D) qRT-PCR was used to analyze the relative expression levels of GOT1, GOT2, MDH1, and MDH2 mRNAs in RA-FLSs (n = 3). (E) PcTX1 reversed the pH 6.8 medium-induced increase in NAD, NADH, and NAD+ levels in RA-FLSs (n = 3). (F) Schematic diagram of AOA inhibition of malate shuttle. (G) Seahorse energy metabolism analyzer detects OCR in RA-FLSs (n = 3). (H) AOA inhibits exogenous malic acid-induced elevation of ATP levels in RA-FLSs (n = 3). (I, J) AOA inhibited migration and invasion of RA-FLSs induced by pH 6.8 medium or exogenous malic acid (n = 3). (K, L) Western blot analysis of N-cadherin and MMP3 protein expression in RA-FLSs (n = 3). The data are presented as the means ± SEM, with the data being analyzed by unpaired two-tailed t-test (two groups) or one-way ANOVA (multiple groups). (*p < 0.05, **p < 0.01).

To investigate the mechanism underlying enhanced mitochondrial respiration in RA-FLSs induced by extracellular acidification, we analyzed differential metabolites in RA-FLSs treated with ASIC1a-regulated pH 6.8 medium. Notably, malic acid, a key metabolite in central carbon metabolism and the TCA cycle, was elevated in RA-FLSs under pH 6.8 conditions, and this increase was reversed by shASIC1a (Figure S8A-B). Exogenous malic acid enhanced RA-FLSs migration and invasion, along with significant increases in N-cadherin and MMP3 expression, as shown by Transwell and Western blot analysis (Figure 5A and Figure S8D). We then explored how ASIC1a mediates the effects of malic acid on mitochondrial respiration. GEO database analysis of synovial tissue revealed that malate-related enzymes, including malic enzyme (ME) and malate dehydrogenase (MDH), as well as enzymes involved in the aspartate-malate shuttle, were significantly upregulated in RA synovium compared to healthy controls (Figure S8E), suggesting abnormal malate metabolism in RA. Notably, MDH1, an NAD oxidoreductase, was particularly elevated in RA synovium. Moreover, metabolomics data showed reduced NAD levels upon ASIC1a knockdown (Figure 5B), hinting at a connection between MDH1 and ASIC1a-mediated metabolic changes. Based on these findings, we focused on MDH1 and its role in regulating NADH shuttle.

HEK293T cells have been treated with pH 6.8 medium to obtain sufficient cytoplasmic protein. And Western blot analysis revealed an upregulation of MDH1 expression after 72 h of pH 6.8 medium treatment, which was inhibited by PcTX1 and GSK872 (Figure 5C). qRT-PCR analysis revealed upregulation of aspartate-malate shuttle enzymes, including GOT1, GOT2, MDH1, and MDH2, in RA-FLSs treated with pH 6.8 for 24 h (Figure 5D). Intracellular NAD and NADH content further confirmed the involvement of ASIC1a/RIPK3 in MDH1-mediated NADH shuttle. Specifically, pH 6.8 treatment increased NAD, NADH, and NAD^+^ levels, which were inhibited by PcTX1 and GSK872 (Figure 5E, and Figure S8F-G). To investigate the role of the malate shuttle in pH 6.8-induced mitochondrial respiration, as well as the migration and invasion of RA-FLSs, we used aminooxyacetic acid hemihydrochloride (AOA), a well-known inhibitor of this shuttle (Figure 5F). Extracellular oxygen consumption rate (OCR) measurements showed that, like PcTX1 and GSK872, AOA significantly inhibited the pH 6.8-induced increase in mitochondrial respiration in RA-FLSs (Figure 5G and Figure S8H). AOA also inhibited malic acid-induced ATP synthesis in RA-FLSs (Figure 5H). Further Transwell and Western blot analyses demonstrated that AOA suppressed the pH 6.8- and malic acid-induced enhancement of RA-FLSs migration and invasion (Figure 5I-L). These results suggest that extracellular acidification promotes mitochondrial respiration and ATP production in RA-FLSs via the ASIC1a/RIPK3-mediated malate shuttle, ultimately facilitating migration and invasion.

### ASIC1a/RIPK3 enhances malate shuttle-mediated mitochondrial respiration via aerobic respiration and glutaminolysis

**Figure 6:**
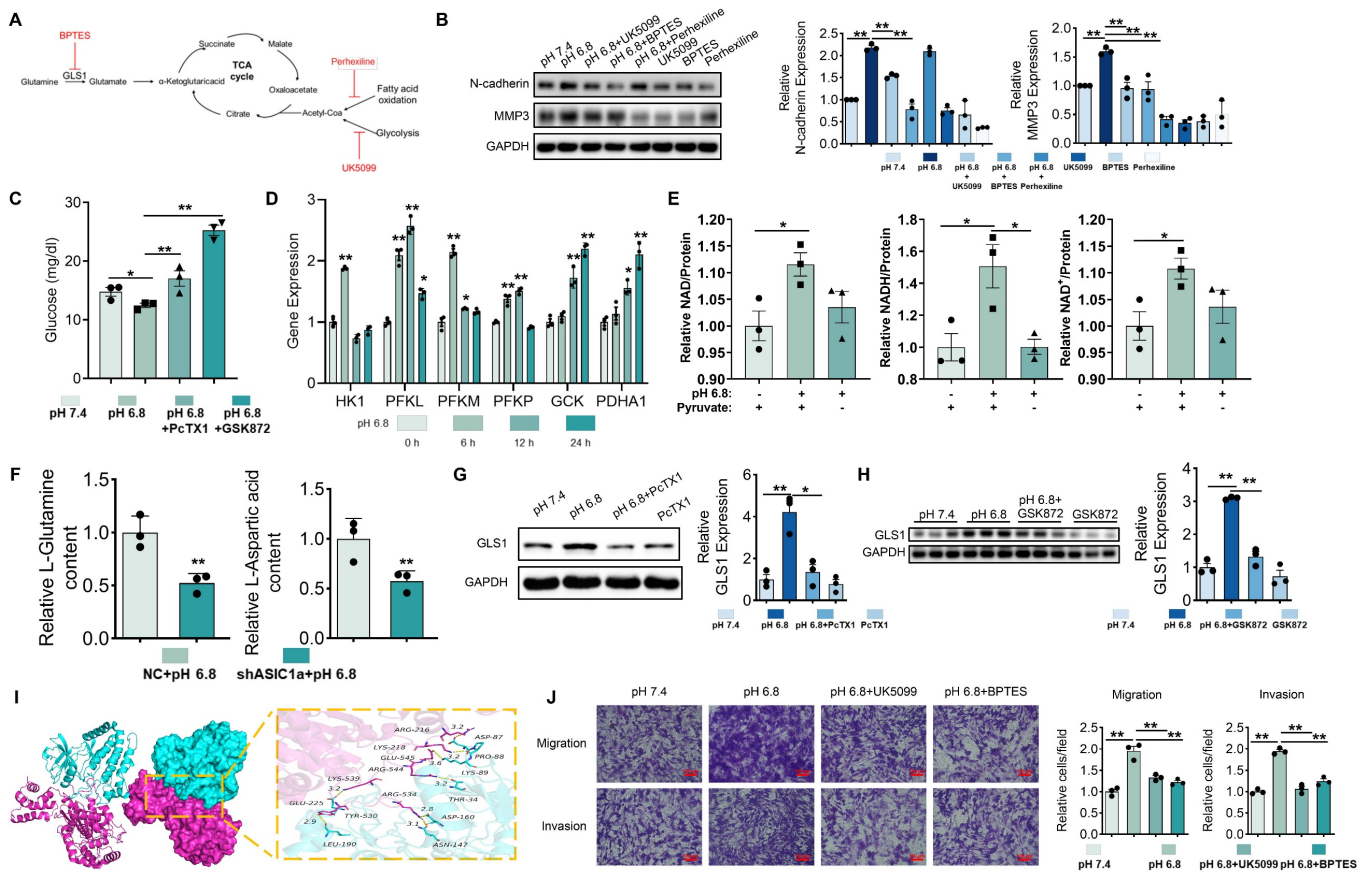
**Glucose metabolism and glutaminolysis promote migration and invasion of RA-FLSs.** (A) Schematic diagram of inhibitor action. (B) Western blot analysis of N-cadherin and MMP3 protein expression in RA-FLSs (n = 3). (C) Glucose content RA-FLSs (n = 3). (D) qRT-PCR analyzed the relative expression levels of HK1, PFKL, PFKM, PFKP, GCK, and PDHA1 mRNAs in RA-FLSs (n = 3). (E) NAD, NADH, and NAD+ content in RA-FLSs (n = 3). (F) Glutamine and aspartate content in RA-FLSs. (G, H) Western blot analysis of GLS1 protein expression in RA-FLSs (n = 3). (I) 3D binding structure of RIPK3 and GLS1 determined via molecular modeling and docking studies. (J) Transwell assayed the effect of the migratory and invasive capacities of RA-FLSs (n = 3). The data are presented as the means ± SEM, with the data being analyzed by one-way ANOVA. (*p < 0.05, **p < 0.01).

We next investigated the metabolic sources contributing to the increased malate levels. Glucose and glutamine are primary carbon and energy sources for mammalian cells, and it has been suggested that supplementation with glucose and glutamine promotes the binding of GOT2 and MDH2, thereby enhancing malate shuttle activity 36. Additionally, fatty acid oxidation may stimulate malate shuttle activity by increasing acetyl-CoA levels 37. To test these hypotheses, we used BPTES, Perhexiline, and UK5099 to inhibit glutaminolysis, fatty acid oxidation, and mitochondrial pyruvate transport, respectively (Figure 6A). Western blot analysis showed that, unlike perhexiline, which only reversed MMP3 expression, UK5099 and BPTES simultaneously reversed the pH 6.8-induced upregulation of both N-cadherin and MMP3 (Figure 6B). This finding suggests that both aerobic glucose metabolism and glutaminolysis exert more prominent roles in driving extracellular acidification-induced migration and invasion in RA-FLSs. We also observed reduced glucose levels in pH 6.8-treated RA-FLSs, but inhibition of ASIC1a and RIPK3 restored glucose depletion (Figure 6C). qRT-PCR analysis revealed that pH 6.8 exposure for 6 h upregulated key glycolytic enzymes (HK1, PFKL, PFKM, PFKP, and GCK), indicating enhanced glycolysis during early extracellular acidification (Figure 6D). Concurrently, PDHA1 levels increased under pH 6.8 treatment (Figure 6D). PDHA1 is crucial for converting pyruvate into acetyl-CoA for mitochondrial entry, favoring NADH production. To investigate this further, we performed pyruvate deprivation experiments. When RA-FLSs were treated with pyruvate-free pH 6.8 medium, NAD, NADH, and NAD^+^ levels did not significantly increase (Figure 6E). However, adding pyruvate to the pH 6.8 medium significantly elevated NAD, NADH, and NAD^+^ levels in RA-FLSs (Figure 6E), suggesting that glycolysis supplies the carbon and NADH necessary for malate shuttle via pyruvate conversion and entry into the TCA cycle.

Metabolomic analysis showed that ASIC1a knockdown in the pH 6.8-treated group suppressed aspartate and glutamine levels (Figure 6F), supporting the notion that ASIC1a knockdown inhibits glutaminolysis and malate shuttle activity. Moreover, upon treatment with pH 6.8, the expression level of GLS1, which serves as a pivotal rate-limiting enzyme in the process of glutaminolysis, was markedly elevated in RA-FLSs. Notably, this upregulated effect could be effectively reversed by the application of PcTX1 and GSK872 (Figure 6G-H). Molecular docking identified multiple potential binding sites between RIPK3 and GLS1 (Figure 6I). Inhibition of glutaminolysis also reversed the pH 6.8-induced increase in OCR in RA-FLSs (Figure 5G and S8H). Additionally, Transwell assays showed that inhibition of mitochondrial pyruvate transport by UK5099 and glutaminolysis by BPTES suppressed the migration and invasion of RA-FLSs induced by pH 6.8 (Figure 6J). These findings suggest that extracellular acidification enhances RA-FLS migration and invasion by promoting aerobic glucose metabolism and glutaminolysis, which provide key carbon sources for malate shuttling.

### Blocking of ASIC1a and RIPK3 attenuate arthritis progression in a CIA model

**Figure 7:**
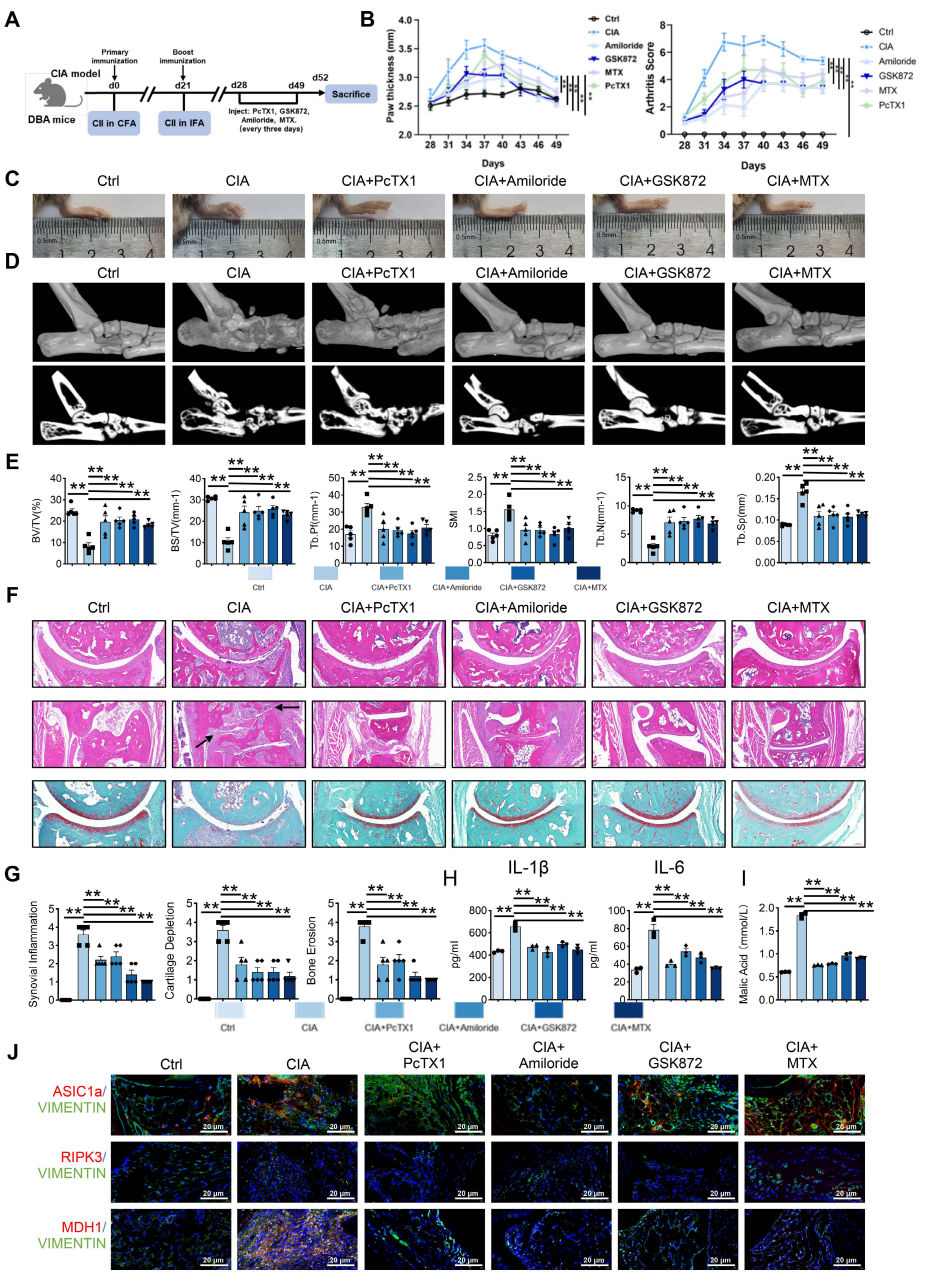
** Blockade of ASIC1a and RIPK3 attenuates the progression of RA in the CIA model.** (A) ASIC1a and RIPK3 inhibitors mitigate arthritis in CIA mice (n = 6).(B, C) Blockade of ASIC1a and RIPK3 significantly inhibits joint swelling, as well as paw thickness and arthritis index in CIA mice (n = 6). (D, E) Micro-CT analysis for the ankles of mice in the indicated groups and quantitative analysis (n = 5). (F) H&E staining shows that the blockade of ASIC1a and RIPK3 significantly inhibits the overall pathogenic destruction, as well as synovial proliferation, migration, and invasion in the joint of CIA mice (black arrows). Safranin O staining demonstrates that ASIC1a and RIPK3 inhibition significantly represses the destruction of bone and cartilage in the joint of CIA mice (n = 5). (G) Histological analysis of synovial inflammation scored, Bone erosion score, and cartilage depletion (n = 5). (H, I) Blockade of ASIC1a and RIPK3 alleviates CIA-induced systemic inflammation and the level of malic acid. (J) Immunofluorescence analysis of ASIC1a, RIPK3 and MDH1 expression in the synovium of CIA model. The data are presented as the means ± SEM, with the data being analyzed by one-way ANOVA or two-way ANOVA. (*p < 0.05, **p < 0.01).

To investigate the regulatory roles of ASIC1a and RIPK3 in arthritis, we treated CIA mice with the ASIC1a-specific inhibitor PcTX1, the non-specific inhibitor Amiloride, and the RIPK3-specific inhibitor GSK872 (Figure 7A). As expected, inhibition of ASIC1a and RIPK3 significantly attenuated arthritis development in the CIA model (Figure 7B). Specifically, PcTX1, Amiloride, and GSK872 reduced joint swelling and the arthritis index (Figure 7B-C). Micro-CT analysis further showed that these inhibitors alleviated arthritis-induced bone damage (Figure 7D-E).

Histological staining (H&E, Safranin O, TRAP) revealed reduced synovial proliferation, invasion, cartilage destruction, and osteoclast formation following inhibitor treatment (Figure 7F-G and Figure S9A). Serum inflammatory assays also indicated that inhibition of ASIC1a and RIPK3 alleviated systemic inflammation (Figure 7H and Figure S9B). Notably, serum malic acid levels were significantly elevated in the CIA model, and this increase was reversed by inhibition of ASIC1a and RIPK3 (Figure 7I). Additionally, immunofluorescence analysis showed reduced expression of MDH1, N-cadherin, and MMP3 in the synovium following inhibition of ASIC1a and RIPK3 (Figure 7J and Figure S10A-B). These results suggest that inhibition of ASIC1a and RIPK3 attenuates RA progression in the animal model, likely through MDH1-mediated malic acid metabolism.

## Conclusion

Extracellular acidification, resulting from lactic acid accumulation due to elevated aerobic glycolysis in RA-FLSs, is a hallmark of RA 10. However, it remains unclear how this acidification alters the metabolic profile of RA-FLSs to promote migration and invasion. In this study, we show that mitochondrial respiration in RA-FLSs treated with acidic medium is significantly enhanced, providing the bioenergy needed for migration and invasion. This increase in mitochondrial respiration is closely linked to the malate shuttle, which supplies NADH for mitochondrial function. In vitro, we found that ASIC1a/RIPK3 regulates this enhanced mitochondrial respiration through the malate shuttle in acidic environment. In vivo, we confirmed the roles of ASIC1a and RIPK3 in RA progression. Mechanistically, ASIC1a promotes MDH1-mediated malate shuttle activity, which, through its interaction with RIPK3, drives RA-FLSs migration and invasion—independent of its ion channel function. These findings highlight ASIC1a/RIPK3-driven metabolic reprogramming as a key mechanism by which RA-FLSs acquire a migratory and invasive phenotype. Thus, targeting the ASIC1a/RIPK3 axis offers a promising therapeutic strategy for RA prevention and treatment.

The pathogenesis of RA involves interactions between synovial fibroblasts, chondrocytes, and immune cells such as macrophages 38. The RA joint microenvironment is typically acidified 39, 40, and ASIC1a, an extracellular sensor, is activated under inflammatory and acidic conditions, contributing to various pathological processes 19. Our previous studies showed that ASIC1a mediates chondrocyte death and promotes synovial lesions 17, 19. Recent research indicates that ASIC1a also regulates mitochondrial respiration and ATP synthesis under acidic conditions 29, 30. Acidification enhances fatty acid oxidation, providing acetyl-CoA for the TCA cycle, supporting mitochondrial respiration 41. Mark et al. showed that an acidified environment promotes autocrine TGFβ2 signaling, enhancing lipid droplet formation and ATP synthesis 42. Additionally, acidification promotes mitochondrial fusion, alters cristae architecture, and reorganizes mitochondria to enhance respiration and ATP production 28. These adaptations help tumor cells meet increased bioenergetic demands when transitioning to an invasive phenotype. Here, we show that extracellular acidification (pH 6.8) enhances mitochondrial respiration and ATP production in RA-FLSs, supporting their bioenergetic needs. Inhibition of mitochondrial respiration with rotenone suppressed migration and invasion of RA-FLSs under acidic conditions, consistent with changes observed in RA synovium. GEO database analysis revealed upregulation of mitochondrial respiration-related genes in RA synovium. These findings suggest that the acidified microenvironment promotes RA-FLSs by enhancing oxidative phosphorylation (OXPHOS). In vivo, blocking ASIC1a reversed acidification-induced mitochondrial respiration and attenuated RA progression, highlighting its critical role in driving disease. Notably, at pH 6.8, RA-FLSs did not show signs of apoptosis or reduced viability, as evidenced by the absence of caspase-3 and Annexin V/PI staining. This suggests that mild extracellular acidification promotes metabolic reprogramming and invasiveness without triggering cell death. One potential explanation is the difference in pH levels—while extreme acidification (e.g., pH 6.0) is known to induce apoptosis or necrosis, the moderate acidity used in our model (pH 6.8) may fall below the threshold required to initiate cell death signaling. Alternatively, RA-FLSs may develop intrinsic resistance mechanisms to survive and function in the chronically inflamed and mildly acidic synovial microenvironment. In addition to HCl-induced acidification, we used sodium lactate to better mimic the physiological acidification of RA synovial fluid. Treatment with 10 mM lactate lowered the medium pH to ~6.8 and similarly promoted RA-FLSs migration, invasion, and upregulation of N-cadherin and MMP3, indicating a more aggressive phenotype (Figure S11). Emerging evidence suggests that lactate not only acidifies the microenvironment but also serves as a metabolic substrate that fuels mitochondrial respiration 43. Thus, lactate may enhance RA-FLSs aggressiveness through both pH-dependent and metabolic effects. Notably, lactate-induced acidification can also activate ASIC1a, further implicating this ion channel in metabolic reprogramming 29. To specifically isolate the effect of extracellular pH from lactate metabolism, we ultimately used HCl to construct the acidification model. This allowed us to focus on the role of acidic pH per se, independent of lactate's potential as a metabolic fuel. By comparing the two models, we show that both promote RA-FLS invasiveness, likely via a shared ASIC1a-OXPHOS pathway, while lactate may also provide distinct metabolic inputs.

Malic acid is a critical component of the malate-aspartate shuttle, essential for mitochondrial function. The electron transport chain (ETC) requires sufficient NADH for mitochondrial respiration; however, NADH cannot directly cross the mitochondrial membrane. In this context, MDH1 catalyzes malate to produce NADH, which is oxidized to NAD and enters the mitochondria. NAD (and NADH) is crucial for mitochondrial function, and synovial metabolomics studies in RA have shown increased Nicotinamide Phosphoribosyltransferase (NAMPT), which helps maintain NAD levels 27. MDH1-mediated malate shuttle to maintain NAD and NADH homeostasis to promote tumor cell survival and metastasis in has been extensively studied. In cells with impaired mitochondrial function, MDH1 promotes migration and invasion by supporting the cytoplasmic reductive carboxylation of glutamine and maintaining NADH circulation 44. Additionally, MDH1 enhances pancreatic ductal adenocarcinoma's resistance to oxidative stress and supports cell proliferation and tumor growth 45. Furthermore, MDH1 has been shown to be activated in acidic extracellular environments, where it functions as a metabolic regulator 46. Recent studies have also shown that MDH1 can be a potential therapeutic target for RA. Visnagina combined with methotrexate can exert anti-inflammatory effects by inhibiting the activity of MDH1 and alleviate the progression of RA 47, but the specific mechanism is unknown. Herein, we found that extracellular acidification increased NAD and NADH levels in RA-FLSs, which was inhibited by blocking ASIC1a. MDH1 was upregulated under acidic conditions and regulated by ASIC1a, correlating with RA progression. Inhibition of the malate shuttle using AOA reversed the enhanced mitochondrial respiration, migration, and invasion induced by pH 6.8, highlighting its key role in these processes.

Previous studies on ASIC1a-mediated joint degeneration focused on calcium influx 19, but no significant increase in calcium was observed in RA-FLSs under pH 6.8, possibly due to ASIC1a's activation threshold or rapid desensitization 48. Notably, ASIC1a can activate RIPK1 independently of its ion channel activity 49. Our prior work showed that ASIC1a inhibitors suppressed RIPK3 50, but its role in RA-FLSs was unexplored. Our findings reveal that RIPK3 plays an active role in the progression of rheumatoid arthritis rather than being a mere bystander or consequence of inflammation. Immunofluorescence staining of synovial tissues and Western blot analysis confirmed significantly elevated expression of RIPK3 in RA-FLSs compared to normal controls. This observation was further supported by transcriptomic analysis of GEO datasets, which showed a consistent upregulation of RIPK3 in RA synovial tissues. Functionally, RIPK3 was shown to promote the migration and invasion of RA-FLSs in response to extracellular acidification in vitro. Co-immunoprecipitation experiments demonstrated a direct interaction between RIPK3 and ASIC1a under acidic conditions, suggesting that ASIC1a may regulate RIPK3 activity through a channel-independent mechanism. To assess the role of RIPK3 in vivo, we employed both CAIA and CIA models and found that genetic deletion or pharmacological inhibition of RIPK3 significantly alleviated disease severity, accompanied by reduced synovial proliferation and invasiveness. These results suggest that RIPK3 is not only upregulated during RA progression but also contributes directly to synovial aggressiveness and joint pathology. Given that ASIC1a drives RA-FLSs migration via the MDH1-mediated malate shuttle, we investigated RIPK3's involvement. Glucose and glutamine were primary carbon sources for the malate shuttle 36. RIPK3 regulates metabolism by activating PDH to promote aerobic respiration 51 and interacting with GLUD1 to enhance glutamine metabolism 52. In RA-FLSs under pH 6.8, glucose levels rapidly decreased but were restored upon RIPK3 inhibition, accompanied by increased PDHA1 transcription, suggesting enhanced pyruvate-driven respiration. pH 6.8 also increased GLS1 expression, which was suppressed by RIPK3 inhibition, indicating that acidification enhances glucose and glutamine metabolism, fueling the TCA cycle and malate shuttle under RIPK3 regulation. Importantly, although RIPK3 is a known mediator of necroptosis, we did not observe activation of this pathway in RA-FLSs under pH 6.8. Western blot analysis showed no significant increase in MLKL or its phosphorylated form (p-MLKL), suggesting that RIPK3 promotes RA-FLS aggressiveness through metabolic regulation rather than necroptotic signaling. These findings highlight a non-canonical, pro-survival role for RIPK3 in the acidified RA synovial environment.

In conclusion, we have for the first time demonstrated that ASIC1a/RIPK3 functions as a metabolic receptor to promote the migration and invasion of RA-FLSs in acidified microenvironments. Specifically, ASIC1a/RIPK3 enhances mitochondrial respiration and ATP synthesis through activation of the MDH1-mediated malate shuttle, while sustaining shuttle activity by promoting glycolysis, facilitating pyruvate entry into mitochondria, and driving glutaminolysis. In vivo, intervention with ASIC1a and RIPK3 significantly alleviated RA progression, highlighting that targeting ASIC1a and its downstream metabolic pathways may offer a promising therapeutic strategy to control synovial invasion and mitigate RA progression.

## Supplementary Material

Supplementary figures and tables.
