# Supplementary material for: RIPK3 promotes ASIC1a-mediated fibroblast-like synoviocyte migration and invasion via malate shuttle-driven mitochondrial respiration in rheumatoid arthritis
Source: Theranostics. 2025 Aug 11;15(17):8719–37. doi: 10.7150/thno.113974 (PMC12439152; doi:10.7150/thno.113974)
Supplement: Supplementary file 1 — Supplementary figures and tables. [file thnov15p8719s1.pdf]

RIPK3 promotes ASIC1a-mediated fibroblast-Like synoviocyte migration and invasion via malate shuttle-driven mitochondrial respiration in rheumatoid arthritis

Weirong Hu[1#], Wang Ke[2#], Yalu Dong[1,2#], Yucai Xu[1,2], Jing Xing[1,2], Jianzhong Zhu[1], Jie Ding[2], Yingjie Zhao[2], Yayun Xu[3], Yuanzhi Cheng[2], Xiaoqing Peng[1], Renpeng Zhou[2,1,4], Wei Hu[2*], and Feihu Chen[1*]*

1. School of Pharmacy; Inflammation and Immune Mediated Diseases Laboratory of Anhui Province, Anhui Institute of Innovative Drugs, Anhui Medical University, Hefei 230032, China.
2. Department of clinical pharmacology, the second affiliated hospital of Anhui Medical University, Hefei, 230601, China.
3. Shenzhen Institute of Translational Medicine, The First Affiliated Hospital of Shenzhen University, Shenzhen Second People's Hospital.
4. Department of Orthopaedics and Rehabilitation, Yale University School of Medicine, New Haven, CT, USA.

#These authors contributed equally

Correspondence: Feihu Chen, School of Pharmacy; Inflammation and Immune Mediated Diseases Laboratory of Anhui Province, Anhui Institute of Innovative Drugs, Anhui Medical University, Hefei 230032, China.

Email: chenfeihu@ahmu.edu.cn

Correspondence: Wei Hu, Department of clinical pharmacology, the second affiliated hospital of Anhui Medical University, Hefei, 230601, China.

Email: huwei@ahmu.edu.cn

Correspondence: Renpeng zhou, Department of clinical pharmacology, the second affiliated hospital of Anhui Medical University, Hefei, 230601, China. Department of Orthopaedics and Rehabilitation, Yale University School of Medicine, New Haven, CT, USA.

Email: zhourenpeng@ahmu.edu.cn

This file includes:

Supplemental Table 1-6

Supplemental Figure 1-11

Supplemental Table

Table S1. Information about synovial tissue sample donors, including rheumatoid arthritis (RA) and normal individuals

Samples	Sex	Age (years)	Disease
1	F	29	N
2	F	49	N
3	F	52	N
4	F	64	N
5	F	60	RA
6	F	71	RA
7	M	62	RA
8	F	55	RA

Abbreviations: RA, rheumatoid arthritis; N, normal; F, female; M, male.

Table S2. Inhibitors and Drug of cell-treatment catalogue

Name	Catalog No.	Source	Application
PcTX1	GC10177	GlpBio	100 nM
GSK872	HY-101872	MCE	5 μM
Malic Acid	S9001	Selleck	10 μM, 20 μM
AOA	HY-107994	MCE	5 mM
Rot	HY-B1756	MCE	0.25 μM, 0.5 μM
UK5099	HY-15475	MCE	10 μM
Perhexiline	HY-B1334A	MCE	5 μM
BPTES	HY-12683	MCE	10 μM

Abbreviations: PcTX1, Psalmotoxin 1; AOA, Aminooxyacetic acid hemihydrochloride; Rot, Rotenone; Perhexiline, Perhexiline maleate.

Table S3. Antibody catalogue

Name	Catalog No.	Source	Application/Dilution
ASIC1a	27235-1-AP	Proteintech	WB/1:1000, IF/1:50
N-cadherin	13116	CST	WB/1:1000
N-cadherin	sc-59987	Santa Cruz	IF/1:50
MMP3	ab52915	Abcam	WB/1:1000
MMP3	sc-374029	Santa Cruz	IF/1:50
GAPDH	ab8245	Abcam	WB/1:10000
RIPK1	ab300617	Abcam	WB/1:1000
RIPK2	ab303554	Abcam	WB/1:1000
RIPK3	ab316957	Abcam	WB/1:1000
p-RIPK3	ab209384	Abcam	WB/1:1000
MDH1	15904-1-AP	Proteintech	WB/1:2000
HSP90	ab203085	Abcam	WB/1:1000
MFN2	ab205236	Abcam	WB/1:1000
ATP5A	ab176569	Abcam	WB/1:1000
MTCO1	13393-1-AP	Proteintech	WB/1:2000
UQCRC2	ab203832	Abcam	WB/1:2000
SDHB	ab175225	Abcam	WB/1:10000
NDUFB8	ab192878	Abcam	WB/1:1000
GLS1	ab317032	Abcam	WB/1:1000
Vimentin	sc-66002	Santa Cruz	IF/1:50

Table S4. Sequences of primers used for qRT-PCR.

gene	Forward Primer	Reverse Primer
SNAI	TCGGAAGCCTAACTACAGCG	AGATGAGCATTGGCAGC
ZEB1	GATGATGAATGCGAGTCAGA	ACAGCAGTGTCTTGTTGT
TWIST	GTCCGCAGTCTTACGAGGAG	GCTTGAGGGTCTGAATCT
Slug	CGAACTGGACACACATACAG	CTGAGGATCTCTGGTTGT
PFKP	AGCTTGCGTCGTGTCACTGA	ATCTCCTCTCGTCCATCG
HK1	TCTTGAACCGCCTGCGTGAT	GGGAATACTGTGGGTGC
GCK	CTCAACTGGACCAAGGGCTT	TCATTCACCATTGCCACC
PFKM	GACTTCTGTGGCACCGATAT	TGGCTCTGGGCAGTGGT
PFKL	CGCTGCTCCTGCCCTCTCC	CACATGCTTCTCATCCGC
LDHA	CAGCCCGATTCCGTTACCTA	TCCACTCCATACAGGCAC
LDHB	GAGGAGCAGAAGGCAGAGG	GCCTCTTCTTCCGCAACT
LDHC	TGCCCGTTTCCGTTACCTAAT	CAGAGCAACACCAGCAA
MDH1	ATCCAGATGTCAACCATGCC	GCCACGCTGCTGCACAG
MDH2	AAGAACCTGGGCATCGGCAA	CGTCACAGCGGCTCACT
GOT1	CCGTCAGTCTTTGCCGAGGT	TATGCTCCCACTCCCAGG
GOT2	TGGTGAGCGTGTAGGAGCCT	GTGTTCAGAATGGCAGC
PDHA1	ACCCTGGAGTCAGTTACCGT	TTGCTGTTCACCATCCTG
GAPDH	GGAGCGAGATCCCTCCAAAA	GGCTGTTGTCATACTTCT

Table S5. Sequences of ASIC1a shRNA

ID	siRNA	shRNA Top strand	shRNA Bottom strand
Con-RNA	TTCTCCGAACGT	GATCCGTTCTCCGAACGTGTCACGTAATTC	AATTGAAAAAATTCTCCGAACGTGTCACGTA
ASIC1a-RNA	CTATGGAAAGTG	GATCCGCTATGGAAAGTGCTACACGTTCTC	AATTCAAAAAACTATGGAAAGTGCTACACGTT

Table S6. Kit catalogue.

Name	Catalog No.	Source
Glucose Assay Kit with O-toluidine	S0201S	Beyotime Biotechnology
Cell and Tissue Lysis Buffer for Glucose Assay	S3062	Beyotime Biotechnology
Enhanced ATP Assay Kit	S0027	Beyotime Biotechnology
NAD^+/NADH Assay Kit with WST-8	S0175	Beyotime Biotechnology
IL-1β ELISA Kit	RX203063M	RUIXIN BIOTECH
IL-6 ELISA Kit	RX203049M	RUIXIN BIOTECH
TNF-α ELISA Kit	RX202412M	RUIXIN BIOTECH
Malic Acid Colorimetric Assay Kit	E-BC-K905-M	Elabscience

Supplemental Figure

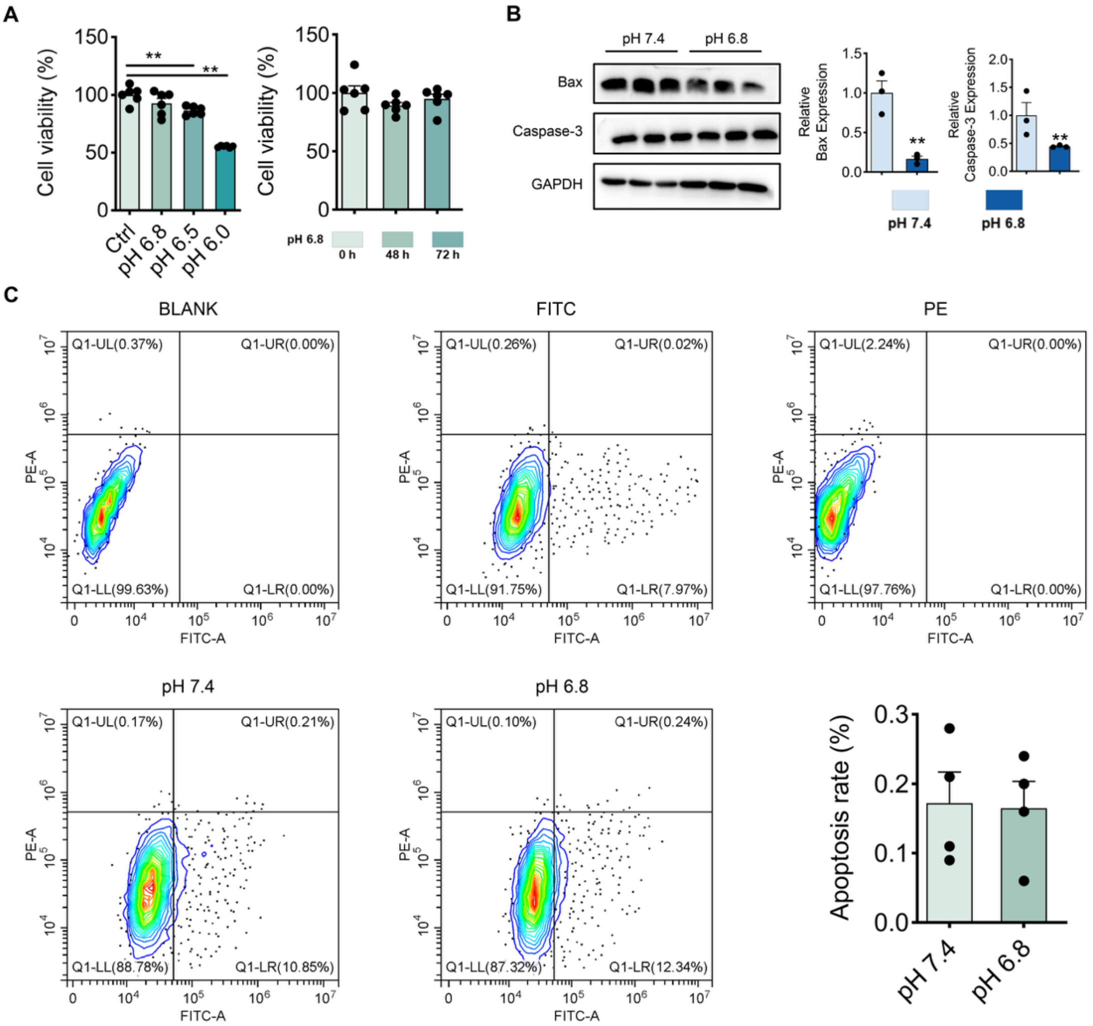

Figure S1. Effects of acidic medium on cell viability and apoptosis in RA-FLSs. (A) Cell viability of RA-FLSs treated with different pH medium for 72 h (left panel) and RA-FLSs treated with pH 6.8 medium at different time points (right panel) (n = 6). (B) Western blot analysis of the differential expression of Bax and Caspase-3 in RA-FLSs after treatment with pH 6.8 medium (n = 3). (C) Apoptosis of cells was assessed by Annexin V-FITC/PI staining and flow cytometry (n = 4). The data are presented as the means ± SEM, with the data being analyzed by unpaired two-tailed t-test (two groups). (*$p < 0.05$, **$p < 0.01$)

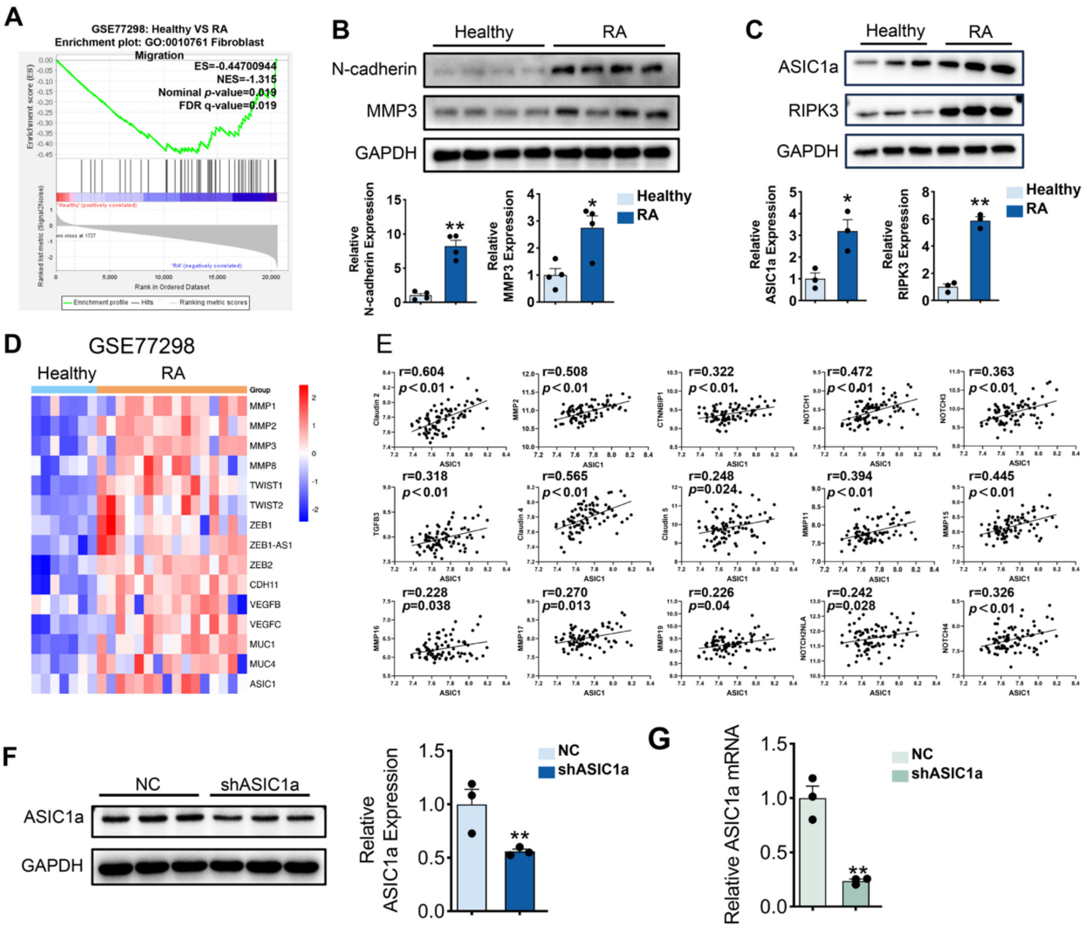

Figure S2. ASIC1a is associated with RA synovial migration and invasion. (A) GSEA enrichment analysis showed that the fibroblast migration pathway was enriched in the synovium of RA patients. (B) Western blot analysis of differential expression of N-cadherin and MMP3 in primary FLSs from healthy donors and RA patients (n = 5). (C) Western blot analysis of differential expression of ASIC1a and RIPK3 in primary FLSs from healthy donors and RA patients (n = 3). (D) Heatmap analysis showing the profile of migration, invasion markers and ASIC1a in the RA versus Healthy group. (E) Correlations between ASIC1a and key genes related to migration and invasion in GSE48780. (F, G) Western blot and qRT-PCR analysis of protein and mRNA expression of ASIC1a in ASIC1a knockdown (n = 3). The data are presented as the means ± SEM, with the data being analyzed by unpaired two-tailed t-test (two groups). (*$p < 0.05$, ***p*

< 0.01)

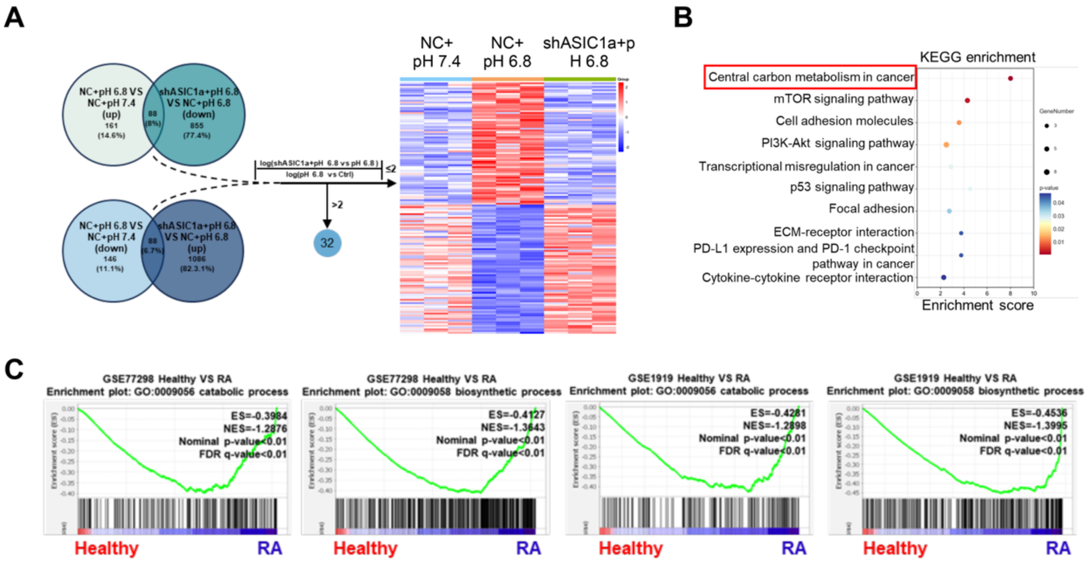

Figure S3. ASIC1a regulates energy metabolism processes in RA-FLSs. (A) Venn diagram showing the overlapping genes between two clusters (NC+pH 6.8 versus NC+pH 7.4, shASIC1a+pH 6.8 versus NC+pH 6.8) to obtain a heatmap of DEGs. (B) GO enrichment analyses showing biological processes associated with these DEGs. (C) GSEA enrichment analysis showed that catabolic and biosynthetic processes associated with increased bioenergy demand were enriched in the synovium of RA patients.

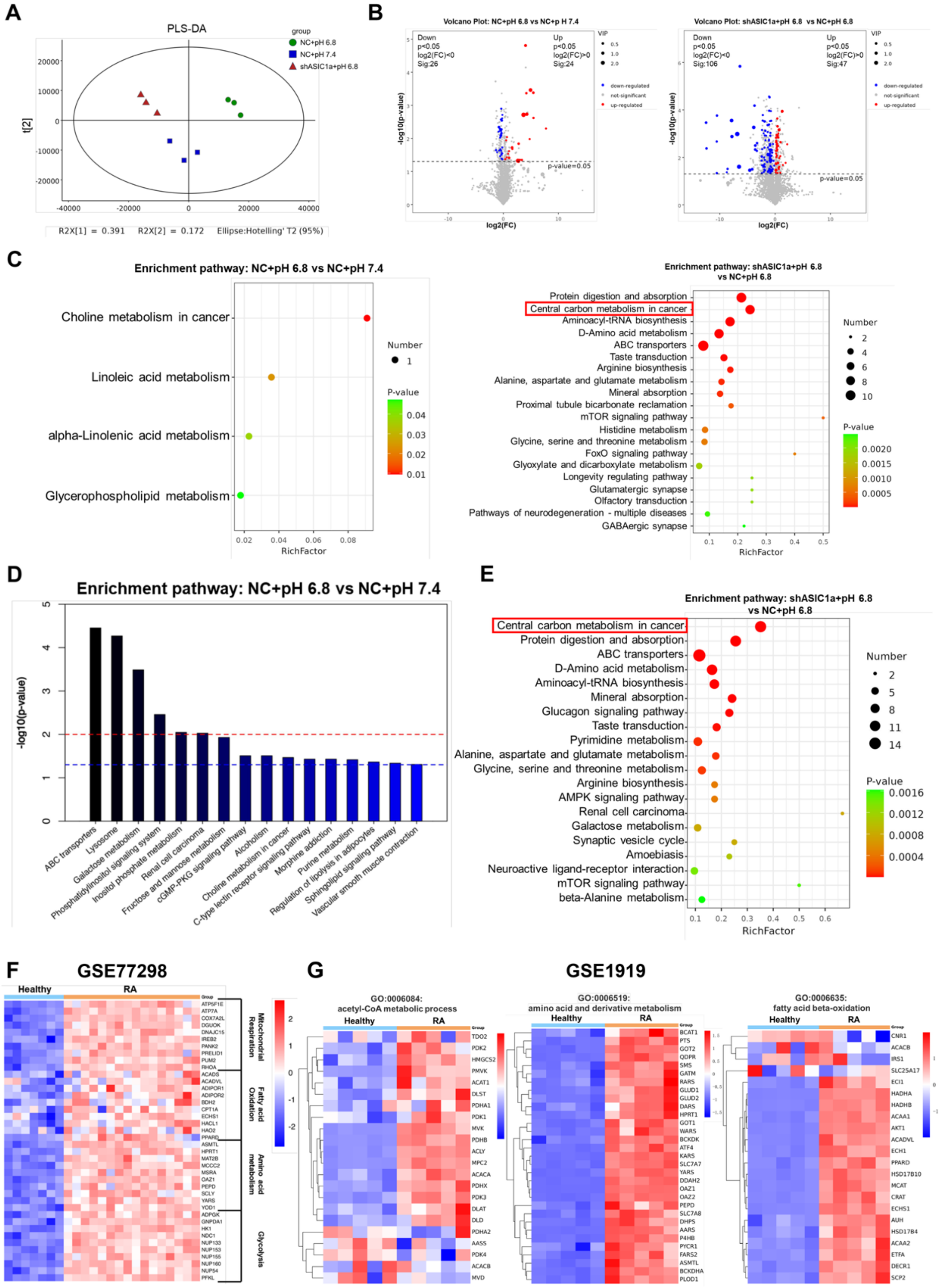

Figure S4. Metabolomic characterization of RA-FLSs by LC-MS/MS. (A) Partial least squares discriminant analysis (PLS-DA) was performed to differentiate the metabolite distribution in the NC + pH 7.4, NC + pH 6.8, and shASIC1a + pH 6.8 groups. (B) GO enrichment analysis showed the metabolic processes of these

differential metabolites. (D, E) GO enrichment analysis of metabolic processes involved in differential metabolites detected by GC MS/MS and LC MS/MS. (F, G) Expression of biomarkers of central carbon metabolism-related pathways in RA synovium, including glycolysis, aerobic respiration, and amino acid metabolism.

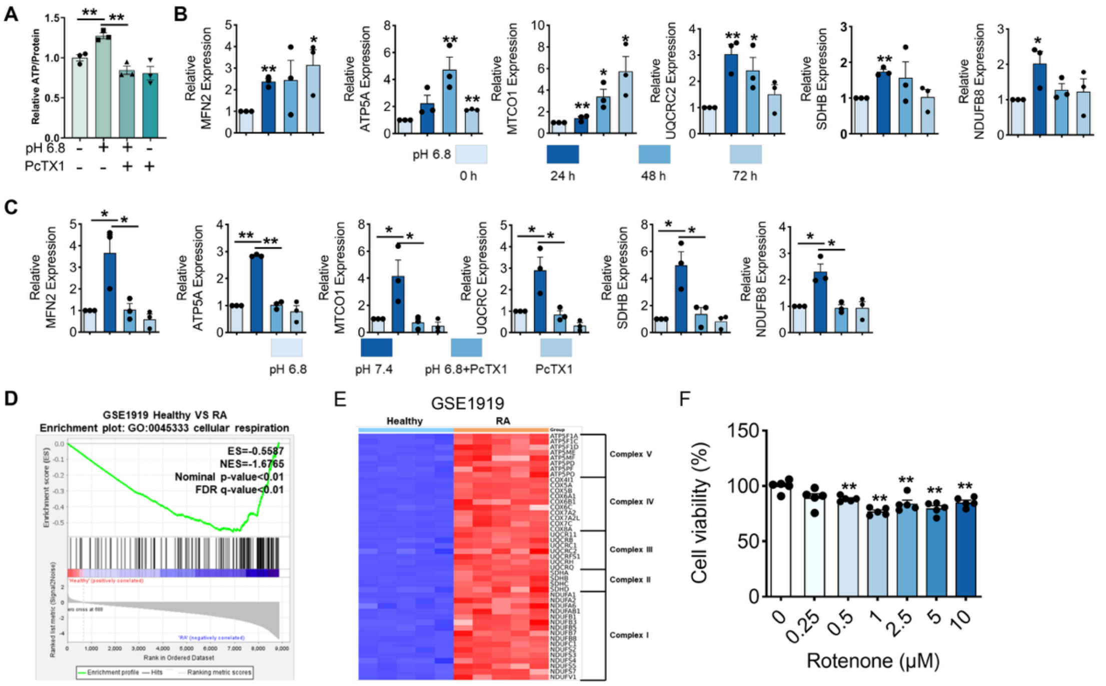

Figure S5. Mitochondrial respiration biomarker expression rises in RA synovium. (A) PcTX1 inhibits the increase in ATP levels induced by pH 6.8 medium (n = 3). Western blot was performed to determine Mfn2 and mitochondrial respiration biomarker levels in pH 6.8 medium-treated RA-FLSs (n = 3). (C) Levels of Mfn2 and mitochondrial respiration biomarkers in RA-FLSs with and without PcTX1 treatment were determined by western blot (n = 3). (D, E) GSEA enrichment analysis and heat map show enhanced mitochondrial respiration in the RA synovium. (F) Cell viability of RA-FLSs treated with Rotenone (n = 6). The data are presented as the means ± SEM, with the data being analyzed by one-way ANOVA. (*$p < 0.05$, **$p < 0.01$)

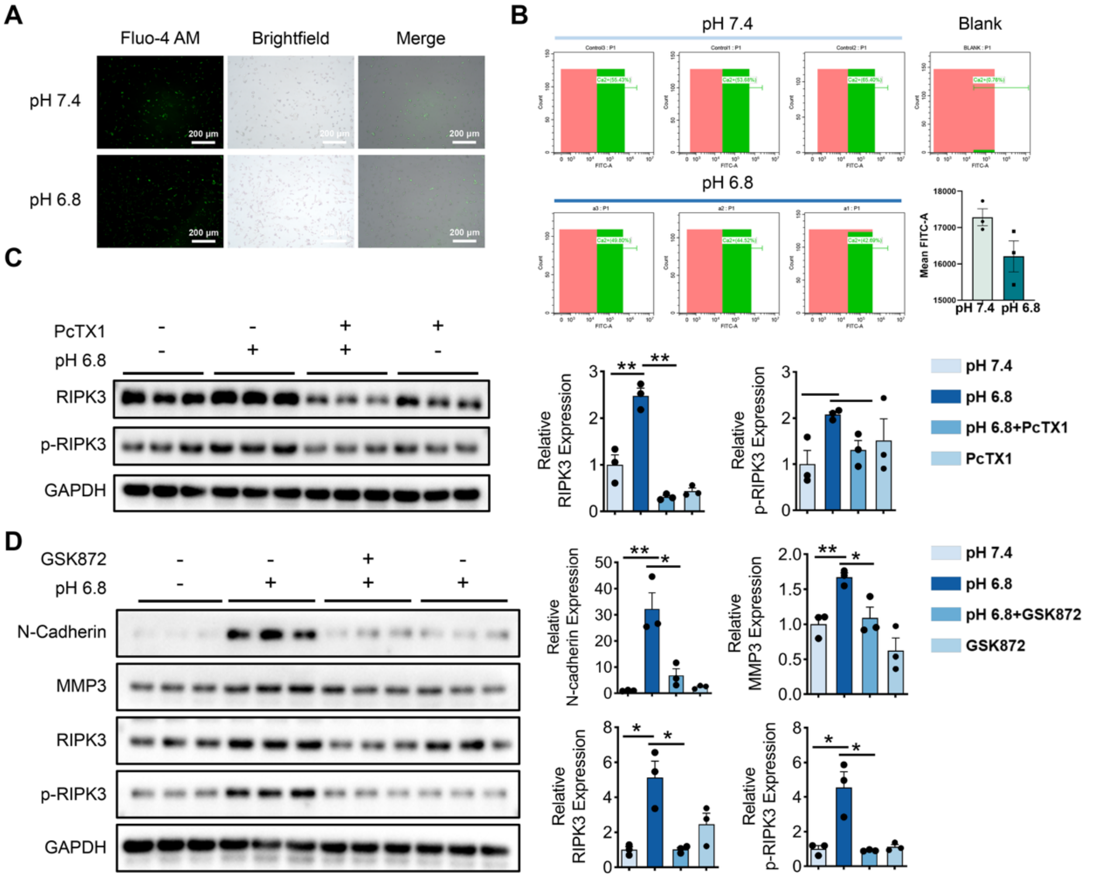

Figure S6. ASIC1a is involved in RA-FLSs migration and invasion through activation of RIPK3. (A, B) Calcium ion abundance in RA-FLSs was measured using Fluo-4 AM (n = 3). (C) Western blot was performed to determine RIPK3, and p-RIPK3 in PcTX1-treated RA-FLSs (n = 3). (D) Western blot analysis of N-cadherin, MMP3, RIPK3 and p-RIPK3 protein expression in RA-FLSs (n = 3). The data are presented as the means ± SEM, with the data being analyzed by one-way ANOVA. (*$p < 0.05$, **$p < 0.01$)

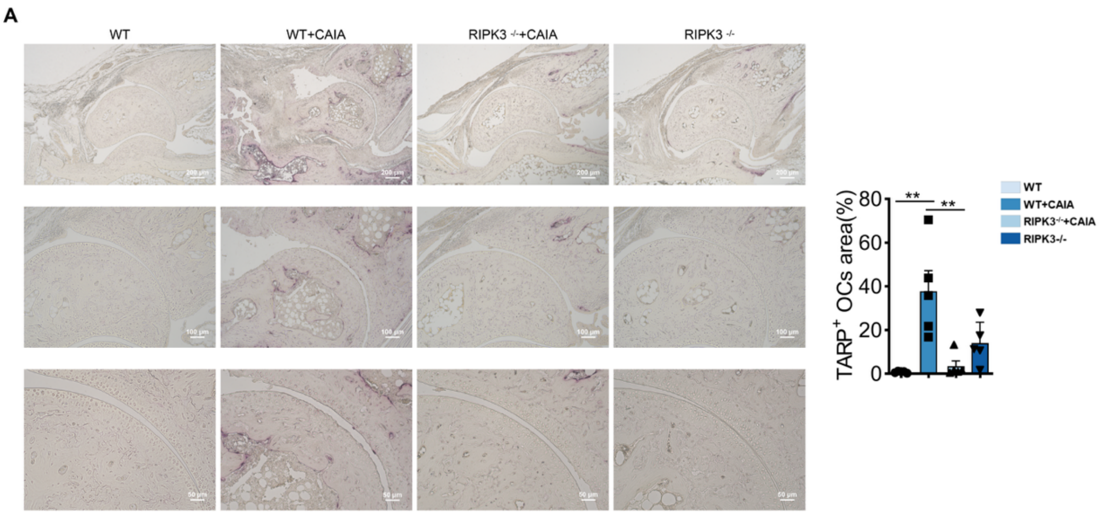

Figure S7. RIPK3 knockout inhibits osteoclast differentiation in the CAIA model.

(A) TRAP staining suggests that RIPK3 knockout inhibits osteoclast differentiation in the joint of CAIA mice (n = 5). The data are presented as the means ± SEM, with the data being analyzed by one-way ANOVA. (*$p < 0.05$, **$p < 0.01$)

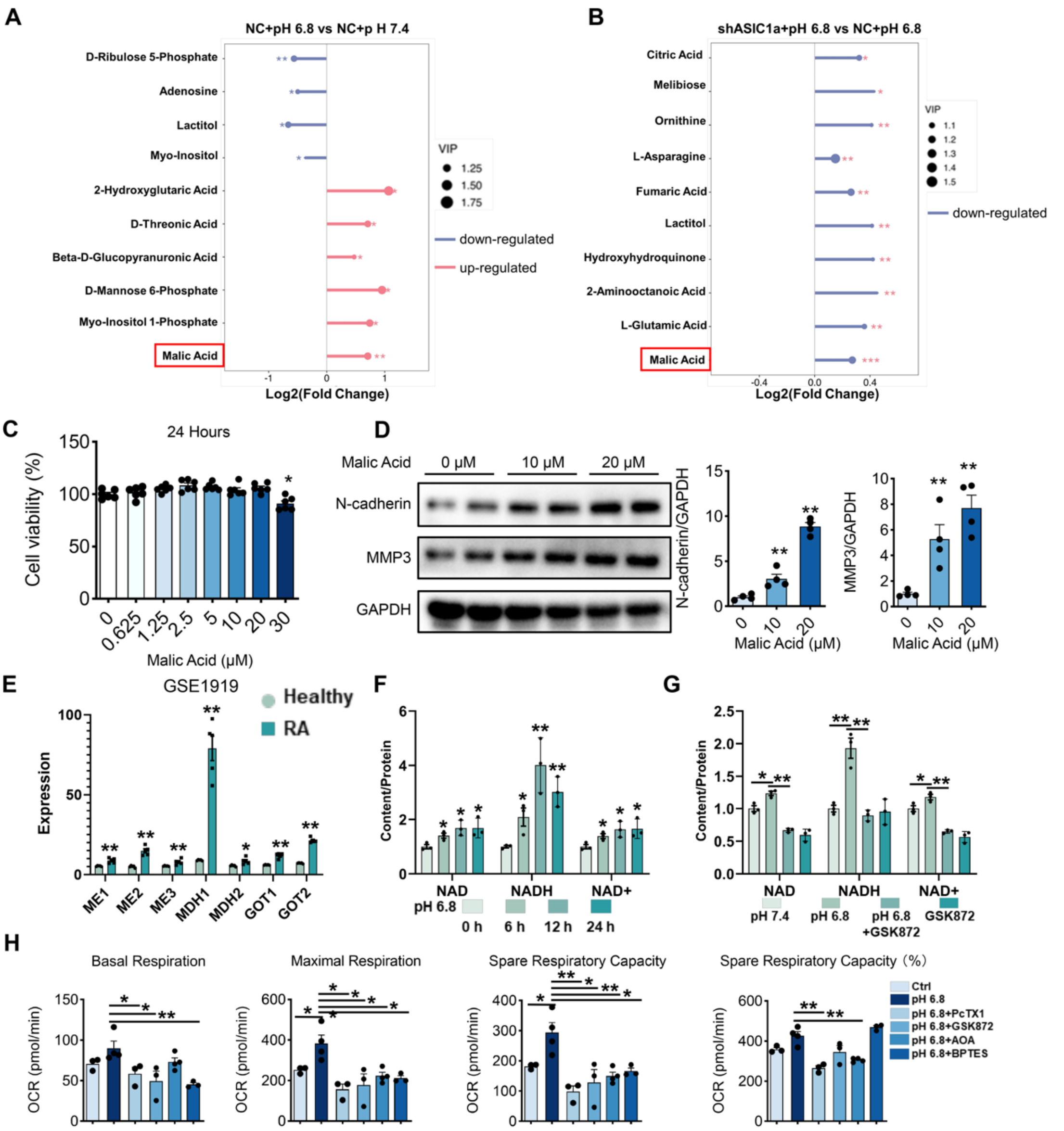

Figure S8. Malic acid promotes mitochondrial respiration and migratory invasion in RA-FLSs. (A) Malic acid content was elevated in pH 6.8-treated RA-FLSs and was reversed after ASIC1a knockdown. (B) Cell viability of RA-FLSs treated with malic acid (n = 6). (C) Western blot analysis of N-cadherin and MMP3 protein expression in RA-FLSs (n = 3). (E) Expression of malate-related metabolizing enzymes in RA (data from GSE1919). (F, G) pH 6.8 medium increased NAD, NADH, and NAD^+ content in RA-FLSs and was inhibited by GSK872 (n = 3). (H) Seahorse energy metabolism analyzer detects mitochondrial respiratory capacity (n = 3). The data are presented as

the means ± SEM, with the data being analyzed by one-way ANOVA. (*$p < 0.05$, **$p < 0.01$)

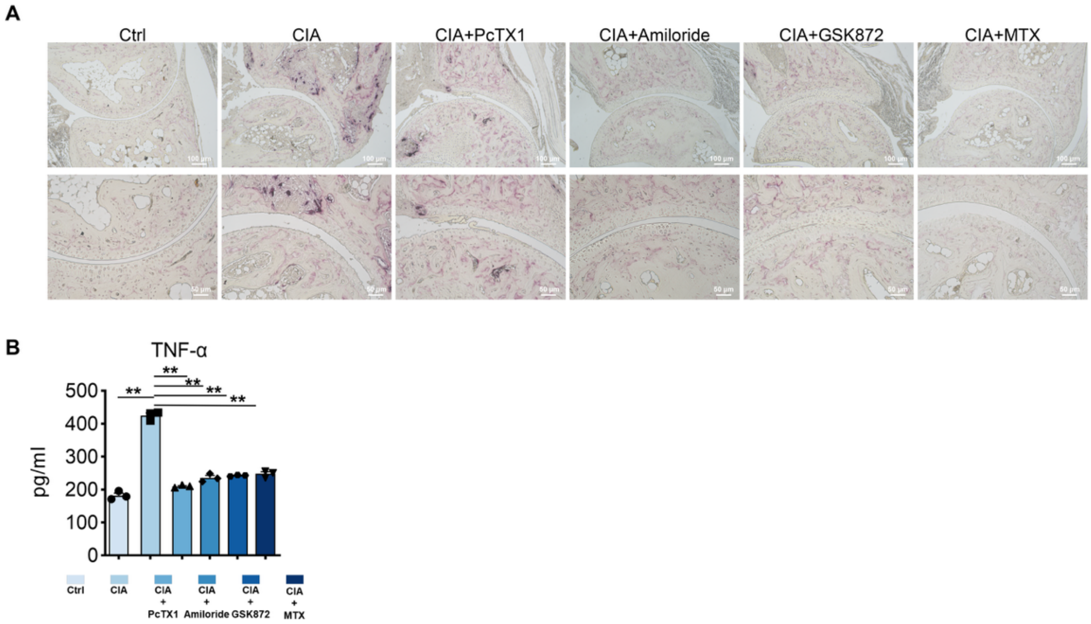

Figure S9. Blockade of ASIC1a and RIPK3 alleviates osteoclast differentiation and systemic inflammation in CAIA. (A) TRAP staining suggests that blockade of ASIC1a and RIPK3 inhibits osteoclast differentiation in the joint of CAIA mice. (B) Blockade of ASIC1a and RIPK3 alleviates CIA-induced TNF-α content (n = 3). The data are presented as the means ± SEM, with the data being analyzed by one-way ANOVA. (*$p < 0.05$, **$p < 0.01$)

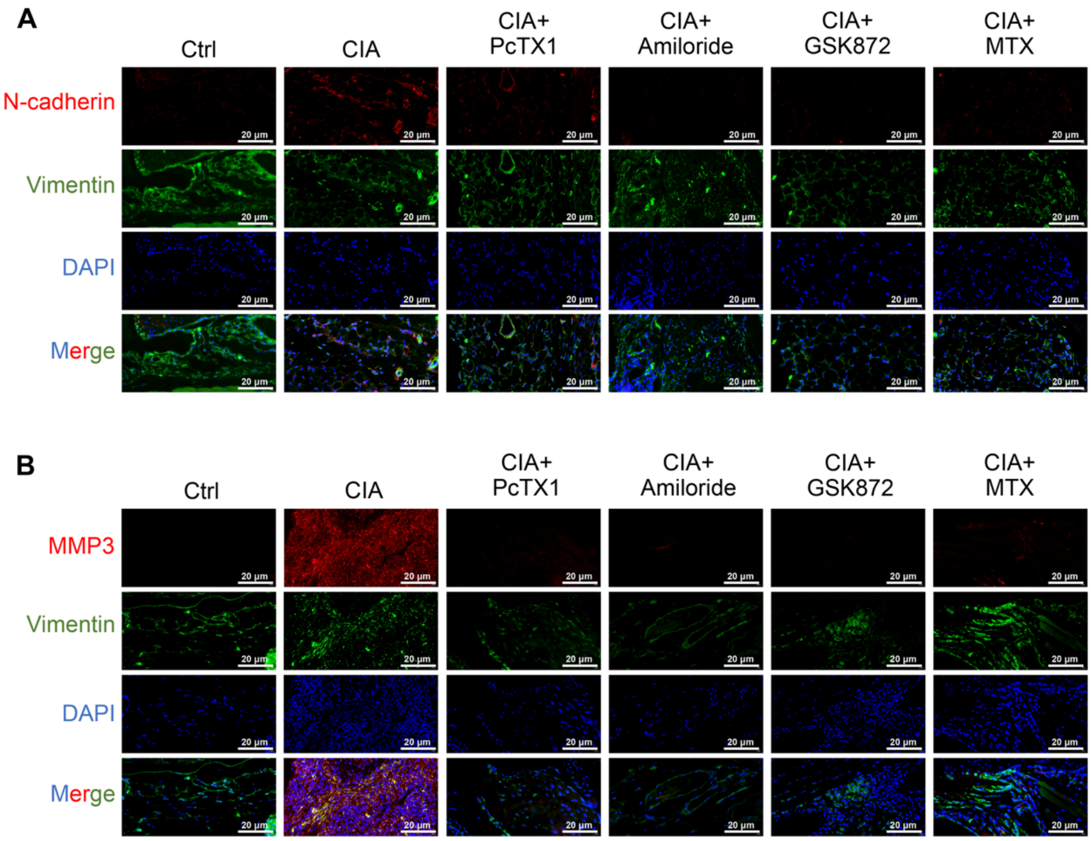

Figure S10. Blockade of ASIC1a and RIPK3 alleviates synovial migration and invasion in CAIA. Immunofluorescence analysis of N-cadherin and MMP3 expression in the synovium of the CIA model.

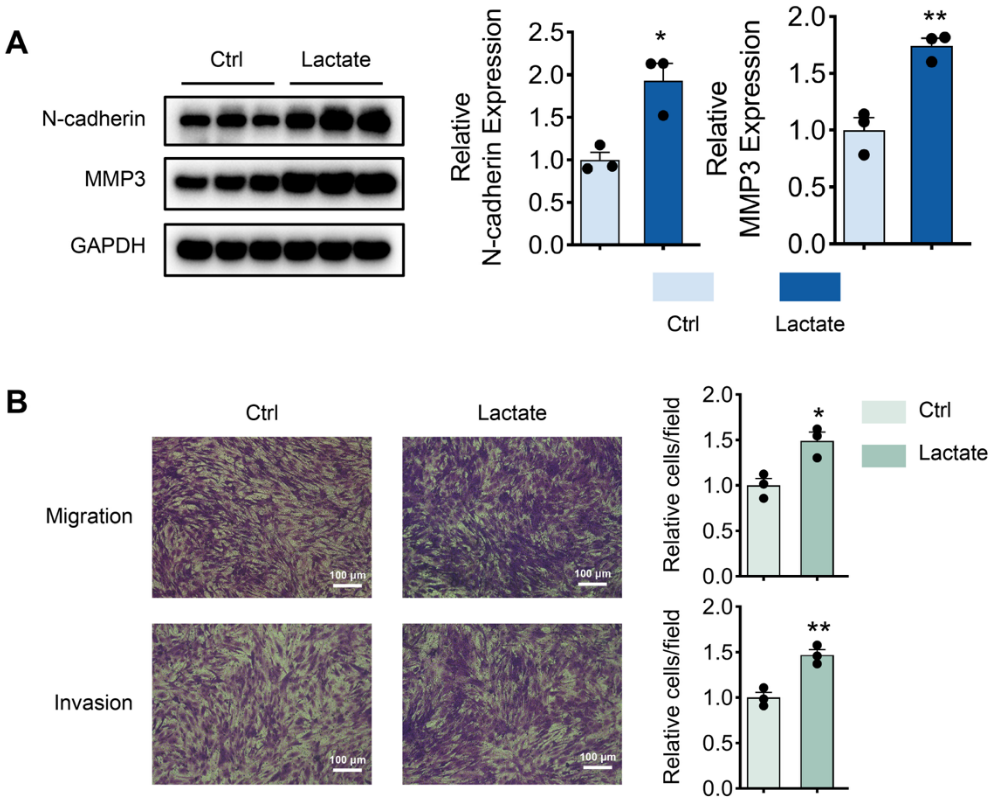

Figure S11. Effects of lactate on the migration and invasion of RA-FLSs. (A) Western blot analysis of the expression levels of N-cadherin and MMP3 in RA-FLSs treated with lactate (n = 3). (B) Transwell assay was performed to evaluate the effects of lactate on the migratory and invasive abilities of RA-FLSs (n = 3). (*p < 0.05, **p < 0.01)
